# Intra-Subtype Variation in Enteroadhesion Accounts for Differences in Epithelial Barrier Disruption and Is Associated with Metronidazole Resistance in *Blastocystis* Subtype-7

**DOI:** 10.1371/journal.pntd.0002885

**Published:** 2014-05-22

**Authors:** Zhaona Wu, Haris Mirza, Kevin Shyong Wei Tan

**Affiliations:** 1 Laboratory of Molecular and Cellular Parasitology, Department of Microbiology, Yong Loo Lin School of Medicine, National University of Singapore, Singapore, Singapore; 2 Department of Dermatology, Yale School of Medicine, New Haven, Connecticut, United States of America; University of Melbourne, Australia

## Abstract

*Blastocystis* is an extracellular, enteric pathogen that induces intestinal disorders in a range of hosts including humans. Recent studies have identified potential parasite virulence factors in and host responses to this parasite; however, little is known about *Blastocystis*-host attachment, which is crucial for colonization and virulence of luminal stages. By utilizing 7 different strains of the parasite belonging to two clinically relevant subtypes ST-4 and ST-7, we investigated *Blastocystis*-enterocyte adhesion and its association with parasite-induced epithelial barrier disruption. We also suggest that drug resistance in ST-7 strains might result in fitness cost that manifested as impairment of parasite adhesion and, consequently, virulence. ST-7 parasites were generally highly adhesive to Caco-2 cells and preferred binding to intercellular junctions. These strains also induced disruption of ZO-1 and occludin tight junction proteins as well as increased dextran-FITC flux across epithelial monolayers. Interestingly, their adhesion was correlated with metronidazole (Mz) susceptibility. Mz resistant (Mz^r^) strains were found to be less pathogenic, owing to compromised adhesion. Moreover, tolerance of nitrosative stress was also reduced in the Mz^r^ strains. In conclusion, the findings indicate that *Blastocystis* attaches to intestinal epithelium and leads to epithelial barrier dysfunction and that drug resistance might entail a fitness cost in parasite virulence by limiting entero-adhesiveness. This is the first study of the cellular basis for strain-to-strain variation in parasite pathogenicity. Intra- and inter-subtype variability in cytopathogenicity provides a possible explanation for the diverse clinical outcomes of *Blastocystis* infections.

## Author Summary

Since it was first described more than a century ago, the question as to whether the protistan parasite *Blastocystis* causes disease or is a commensal of the human gut still remains unresolved. Strain- or subtype-dependent variability in *Blastocystis* pathogenicity has been proposed to contribute to this controversy. Currently, the factors determining this variation are unknown. For seven strains from two clinically relevant *Blastocystis* subtypes, we evaluated their ability to adhere to human intestinal epithelium and to disturb barrier function. We showed that the more adhesive strains exhibited greater pathogenicity. We also observed an inverse correlation between adhesiveness and drug resistance, suggesting that drug resistance might compromise the fitness of the parasite. This is the first study highlighting the important role of adhesion in *Blastocystis* pathogenesis. We conclude that the extensive variation in *Blastocystis* pathogenicity is a plausible factor contributing to the disparate outcomes of *Blastocystis* infections.

## Introduction

*Blastocystis* is a unicellular, genetically diverse protist, phylogenetically placed within the Stramenopiles [1], and it is the only member of this group associated with human pathological changes [2], [3]. It is a species complex comprising 17 subtypes (STs) at least 9 of which are found in humans [4], [5]. The prevalence of this parasite is usually higher in developing countries, ranging from 30% to 50%, and 1.5% to 15% in developed countries [2], [6]. However, some select populations in developed countries may have much higher prevalence [7]. It is frequently reported in human fecal samples from symptomatic patients as well as healthy individuals [8]–[10]. The parasite induces enteritis, manifested in mucous and watery diarrhea, bloating, abdominal pain and/or vomiting [3]. Clinical studies also associate *Blastocystis* with other intestinal and dermatological inflammatory disorders, such as irritable bowel syndrome and urticaria [11]–[14], respectively. Patients immunocompromised due to HIV or cancer are particularly susceptible to infections [15]–[18], suggesting that *Blastocystis* is also an opportunistic pathogen.

Despite being discovered more than 100 years ago [19]–[21], it is difficult to argue the clinical significance and pathogenic potential of *Blastocystis* [21], since infections do not consistently lead to intestinal symptoms [22]–[24]. While a large number of infected individuals present with clinical symptoms [22], [25], asymptomatic carriage of the parasite is also common [26]. Moreover, in symptomatic patients, the duration and severity of symptoms vary from acute enteritis to chronic, mild diarrhea [24], [27]. There is no consensus on the reasons for the observed diverse intestinal symptoms. A number of reports have suggested a strain- or subtype-dependent variation in parasite pathogenicity [1], [24], [27]–[31]. Studies associate ST-1, -4, and -7 with pathological alterations in humans, whereas ST-2 and ST-3 are considered apathogenic [22], [24], [28]. Also, the presence of both pathogenic and apathogenic strains within one subtype has been reported [32], [33]. Yet, the factors determining this variation in pathogenicity across different *Blastocystis* strains have not been resolved. Although recent advances have been made in the knowledge of its molecular and cellular biology [3], [21], [34], [35], as well as pathogenic mechanisms [30], [36], many gaps remain unfilled regarding the pathogenesis of *Blastocystis*. The chain of events leading to parasite-induced pathological changes are largely unknown, the most important early event being *Blastocystis*-enterocyte contact [37].

Extracellular, enteric pathogens have to overcome the intestinal luminal peristaltic flow to remain in the intestine [38], [39]. Adhesion to epithelial cell surfaces is thus a critical step for their infection and effective colonization of the host [40]. Studies have suggested the level of adhesion is directly linked to the virulence properties of pathogens [40], [41]. Highly adhesive strains of *Giardia*, *Entamoeba*, *Trichomonas* and other eukaryotes have been shown to result in more severe damage of the epithelium compared with less adherent strains [42]–[44]. Unlike other luminal parasites, *Blastocystis* is immotile [26]; hence, efficient anchoring to epithelial cells is even more crucial for its survival in the host gut as well as the induction of entero-pathogenesis. The ability of *Blastocystis* to adhere to the intestinal epithelium has not yet been investigated. Clearly, it is important to determine adhesiveness of the parasite with enterocytes across different *Blastocystis* strains and investigate its association with parasite pathogenicity.

Another issue complicating the pathogenic potential of *Blastocystis* is reports of treatment failure [25], [27], [45]–[48]. Although metronidazole is the treatment of choice, physicians are often skeptical about prescribing antibiotics for *Blastocystis* infections due to frequent reports of non-responsiveness to chemotherapy [24]. Strain-to-strain variation within *Blastocystis* in susceptibility to Mz and other antiparasitic agents among *Blastocystis* strains is commonly reported [49], [50], and has been proposed to be the reason for frequent treatment failures in parasite infections [25], [49]. However, from an evolutionary standpoint, mutations associated with drug resistance may impair essential biological functions or impose energy demands on the organism, leading to decreased microbial fitness [51], [52]. Studies of a variety of pathogens, including different species of viruses, bacteria and parasites, indicate that antimicrobial resistance places a toll on the organisms' fitness as well as virulence [53]. A recent study in an intestinal protozoan parasite, *Giardia*, revealed impaired attachment and decreased infectivity in Mz resistant (Mz^r^) strains compared with parental Mz sensitive (Mz^s^) strains [54] and was given as a possible reason for the scarcity of treatment failure in people with symptomatic parasite infections. The interplay among drug resistance, fitness and virulence in *Blastocystis* has never been studied. Considering the frequent reports of treatment failure in humans with symptomatic *Blastocystis* infections, it will be interesting to establish whether drug resistance exerts any effects on the pathogenicity as well as other aspects of parasite fitness.

In the present study, using seven isolates from two clinically important subtypes *Blastocystis* ST-4 and ST-7, we first demonstrated extensive intra- and inter-subtype variability in inducing intestinal barrier dysfunction, and then investigated whether adhesion contributed to this variation. We found that Mz resistance correlates with impairment of parasite adhesion and adhesion-associated cytopathic effects. Additionally, we also showed that Mz resistance was associated with nitrosative stress tolerance, an important factor assisting parasite survival in the gut lumen.

## Materials and Methods

### Culture of Caco-2 colonic epithelial cell line

All *Blastocystis*-host interaction experiments were performed using Caco-2 human colonic cell line (ATCC). Caco-2 stock cultures were maintained in T-75 flasks in a humidified incubator with 5% CO_2_ at 37°C. Cell cultures were grown in Dulbecco's modified Eagle's medium (HyClone) supplemented with 10% heat-inactivated fetal bovine serum (FBS) and 1% each of sodium pyruvate and MEM, antibiotic Penicillin-Streptomycin (Gibco; final concentrations are 1,000 units/mL of penicillin and 1,000 µg/mL of streptomycin). Culture health was evaluated using trypan blue assay; only cultures with >95% viability were used for the experiments. Cells were trypsinzed with 0.25% trypsin-EDTA for subculture and cell seeding. For Western blot analysis, Caco-2 monolayers were grown on 6-well cell culture plates (Corning) until 100% confluency. For immunofluorescence and confocal microscopy, Caco-2 monolayers were cultured on Poly-L-lysine-treated 15 mm glass coverslips placed in standard 24-well culture plates. For epithelial permeability experiments, cells were grown on Millipore transwell filters with PET membranes of a 3 µm pore-size, placed in 24-well tissue culture plates. In order to synchronize cells before experiments, all cultures were serum-starved overnight in antibiotic- and serum-free DMEM.

### Parasite culture

Seven axenized isolates of *Blastocystis* (designated B, C, E, G, H, S-1 and WR-1) were used in the present study (Figure S1). All seven isolates were subtyped previously by small-subunit of ribosomal RNA gene analyses [55]. Isolates B, C, E, G and H were originally recovered from symptomatic patients at the Singapore General Hospital [56] and they all belonged to ST-7 according to recent classification system [57]. Isolates S-1 and WR-1 were isolated from a rat during an animal survey [58] and they belonged to ST-4.

Both ST-4 and ST-7 are well characterized zoonotic *Blastocystis* isolates commonly detected in humans with gastrointestinal symptoms [24]. Stock cultures of all seven isolates were maintained under the same conditions as described previously [49]. In brief, the parasites were maintained in 10 ml of pre-reduced Iscove's modified Dulbecco's medium (IMDM) containing 10% heat-inactivated horse serum in an anaerobic jar (Oxoid) with an AnaeroGen gas pack (Oxoid) at 37°C.

### Ethics statement

All *Blastocystis* isolates used in this study were obtained from an existing collection at the Department of Microbiology of the National University of Singapore (NUS). Human isolates were obtained from patients at the Singapore General Hospital in the early 1990s, before Institutional Review Board was established in NUS. All samples were anonymized.

### Parasite viability assay

The optimized *Blastocystis* viability assay was used to measure 50% inhibitory concentrations (IC50s) of different drugs for the *Blastocystis* strains [49]. Briefly, stock solutions of drugs were prepared in dimethyl sulfoxide (DMSO), diluted in pre-reduced *Blastocystis* culture medium, transferred to 96-well plates and pre-reduced in anaerobic jar for 4 h at 37°C. Parasites were counted and 0.5×10^6^ cells were incubated in each well of a standard 96-well plate with dilutions of different drugs ranging between 0 and 100 µg/ml. The drug concentration range was reduced or increased depending on the results of the first test. A range of parasite counts between zero and 0.5×10^6^ per well were used for viability control. The final DMSO concentration was kept constant at 0.5% in each well. Since the parasite redox activity varies with volume, the final total volume of each well was kept constant at 200 µl. After 24 h of drug exposure, resazurin solution (Sigma) was added to each well at a final concentration of 10% (v/v); 3 h after incubation, under anaerobic conditions at 37°C, fluorescence readings of resazurin were taken at 550-nm excitation and 580-nm emission wave lengths using a Tecan Infinite M200 reader. Values were imported into GraphPad Prism5 software and IC50s were calculated.

### Growth curve

The growth curve of each of the seven isolates was made by culturing over a period of 96 h. Briefly, 5×10^6^ parasites of each isolate were inoculated into IMDM and cultured under anaerobic conditions at 37°C. At each of the five time points (0, 24, 48, 72 and 96 h), parasite pellets were resuspended and cell numbers were counted using appropriate dilutions in a hemocytometer.

### Azocasein assay for cysteine protease activity

Parasite protease activity was determined using an azocasein assay [59]. Briefly, lysates of 4×10^6^ parasites were co-incubated with 2 mM dithiothreitol (DTT) (Sigma) at 37°C for 10 min to activate protease activity. 100 µl of a 5 mg/ml solution of azocasein (Sigma) was prepared in PBS (pH 7.4) and incubated with 100 µl of parasite lysate for 1 h at 37°C. The reaction was stopped by adding 300 µl of 10% trichloroacetic acid (TCA), and samples were incubated on ice for 30 min. Undigested azocasein was removed by centrifugation (5,000×g for 5 min), and the resultant supernatant was transferred to a clean tube containing 500 µl of 525 mM NaOH. Absorbance was measured using a spectrophotometer at 442 nm (Tecan Magellan). PBS was used as a negative control. For inhibition experiment, 2 mM iodoacetamide (IA) were added to the parasite lysate and incubated for 1 h at room temperature (22–24°C) to inhibit the cysteine proteases [60].

### Adhesion assay

One-day-old *Blastocystis* cells were collected and stained with carboxyfluorescein diacetate succinimidyl ester (CFSE; Invitrogen) at a final concentration of 20 µM. Pellets were then centrifuged at 900 g for 10 min to remove the excessive stain and were diluted with pre-warmed (37°C) serum-free culture medium. For each well of Caco-2 cells grown on coverslips in 24-well tissue culture plates, 1.25×10^7^ parasites were added onto Caco-2 cell monolayer and incubated for 1 hour at 37°C. After incubation, cells were washed 5 times with sterile PBS and fixed with 2% formaldehyde for 30 min. The monolayers were then washed and stained with DAPI for 10 min and washed again. All monolayers were mounted onto glass slides using fluorescence mounting media (VECTASHIELD) prior to confocal microscopic examination (Olympus Fluoview FV1000, Olympus, Japan).

### Epithelial permeability

Caco-2 monolayers were grown on Millipore transwell system until they reached confluency and tight junction maturation on day 21. After confirmation of maturation by TER measurement, monolayers were co-incubated with parasite live cells for 24 h. Following co-incubation, epithelial and basolateral compartments were washed twice, followed by addition of 400 µl of warm (37°C) HBSS at the basolateral compartments, and 200 µl of 100 mg/ml FITC-conjugated Dextran 4000 (Sigma) solution in HBSS to apical compartments. After 1 h at 37°C, 300 µl of HBSS was taken from basolateral compartments and was transferred to a black 96-well plate (NUNC) to estimate dextran–FITC flux across monolayers. Fluorescence was measured using an ELISA reader (Tecan Infinite M200) at excitation and emission wavelengths of 492 nm and 518 nm respectively.

### Western blotting

Caco-2 cells were plated at a density of 2×10^4^/cm^2^ on a 6-well culture plate and allowed to reach confluency. After treatments, cell monolayers were then rinsed three times with chilled sterile PBS (pH 7·4) and lysed on ice for 40 min with 150 µl of ice-cold radioimmunoprecipitation assay (RIPA) buffer (150 mMNaCl, 1.0% Triton X-100, 0.5% sodium deoxycholate, 0.1% SDS, 50 mMTris-HCl, pH 8.0), including protease and phosphotase inhibitors for proteins extraction. After centrifugation at 16,000 *g* for 30 min at 4°C, the supernatant was collected for further analysis. Equal amounts of total protein were separated on 10% SDS-polyacrylamide gels and then transferred to a nitrocellulose membrane. After blocking for 2 h in PBS containing 0.1% Tween and 5% (w/v) skim milk, membranes were incubated overnight at 4°C, in primary antibody (1∶3000, mouse anti-ZO-1, anti-Occludin; Zymed). After five washes for 5 min each with PBS-T, the membranes were incubated for 1 h with horseradish peroxidase-conjugated secondary antibody (1∶2000). After another five washes with PBS-T, the membranes were developed using enhanced chemiluminescence reagent (Amersham, Princeton, NJ, USA) to detect proteins.

### Confocal microscopy

Nitric oxide cytotoxicity against *Blastocystis* was investigated by confocal microscopy (Annexin-FITC/PI staining). Live *Blastocystis* were treated for 3 h with a 50 µg/ml concentration of GSNO (S-Nitrosoglutathione), a nitric oxide donor. After drug exposure, the parasites were washed and re-suspended in Annexin V binding buffer (BioVision). Fluorescein isothiocyanate (FITC)-labeled annexin V and propidium iodide (PI) (BioVision) were then added to the cell suspension at the ratio of 1∶100. Imaging of cell suspensions was conducted using a confocal microscope (Olympus Fluoview FV1000, Olympus, Japan). Images were captured using Olympus Fluoview v. 1.6b.

To study tight junction proteins in Caco-2 cells, Caco-2 cells were grown on Poly-L-lysine treated glass coverslips to 100% confluency. After treatment, monolayers of cells were rinsed three times with chilled sterile PBS (pH 7·4), fixed with 2% formaldehyde for 30 min, permeabilized with 0.1% Triton ×100 for 10 min, blocked with 10% FBS for 2 h at room temperature and then incubated with respective primary antibodies to ZO-1 and occludin overnight at 4°C. After five washes for 5 min each with PBS, the monolayer was then incubated with Cy3-conjugated secondary antibodies. Glass coverslips were then mounted onto clean glass slides using fluorescence mounting media (VECTASHIELD) and examined under confocal laser microscopy.

To explore the spatial relationship between *Blastocystis* and Caco-2 monolayers after attachment, Caco-2 monolayers were grown to confluency on glass converslips and were then co-incubated for 1 h with live *Blastocystis* cells. After 1 h incubation, cells were washed 5 times with sterile PBS and fixed with 2% formaldehyde for 30 min, blocked with 10% FBS for 2 h at room temperature and then incubated with legumain antibody-mAb1D5 [35], an in house murine IgM monoclonal antibody [61], and secondary Alexa Fluor® 594 goat anti-mouse IgM (red). Phalloidin-FITC (green) was used to label F-actin of Caco-2 cells and DAPI (blue) for nuclei. All monolayers were mounted on to glass slides using fluorescence mounting media (VECTASHIELD) before confocal microscopic examination.

### Drug preparation

Mz, GSNO and NaNO_2_ were purchased from Sigma. Stock solutions of each compound to be tested were prepared fresh in DMSO. For drug sensitivity determination, stock solutions were diluted in prereduced *Blastocystis* medium and transferred to 96-well plates and pre-reduced again for 4 h. The final DMSO concentration was kept constant at 0.5%.

### Statistical analysis

The ANOVA test was used to confirm the statistical significance of the results. Correlation analyses between Mz resistance and fitness parameters (i. e. attachment, permeability increase and nitric oxide tolerance) were performed using the Pearson correlation method in GraphPad Prism 6 (GraphPad Software Inc., San Diego, CA, USA).

## Results

### *Blastocystis* exhibits intra- and inter-subtype variation in ability to induce epithelial permeability increase in human epithelium

**Figure 1:**
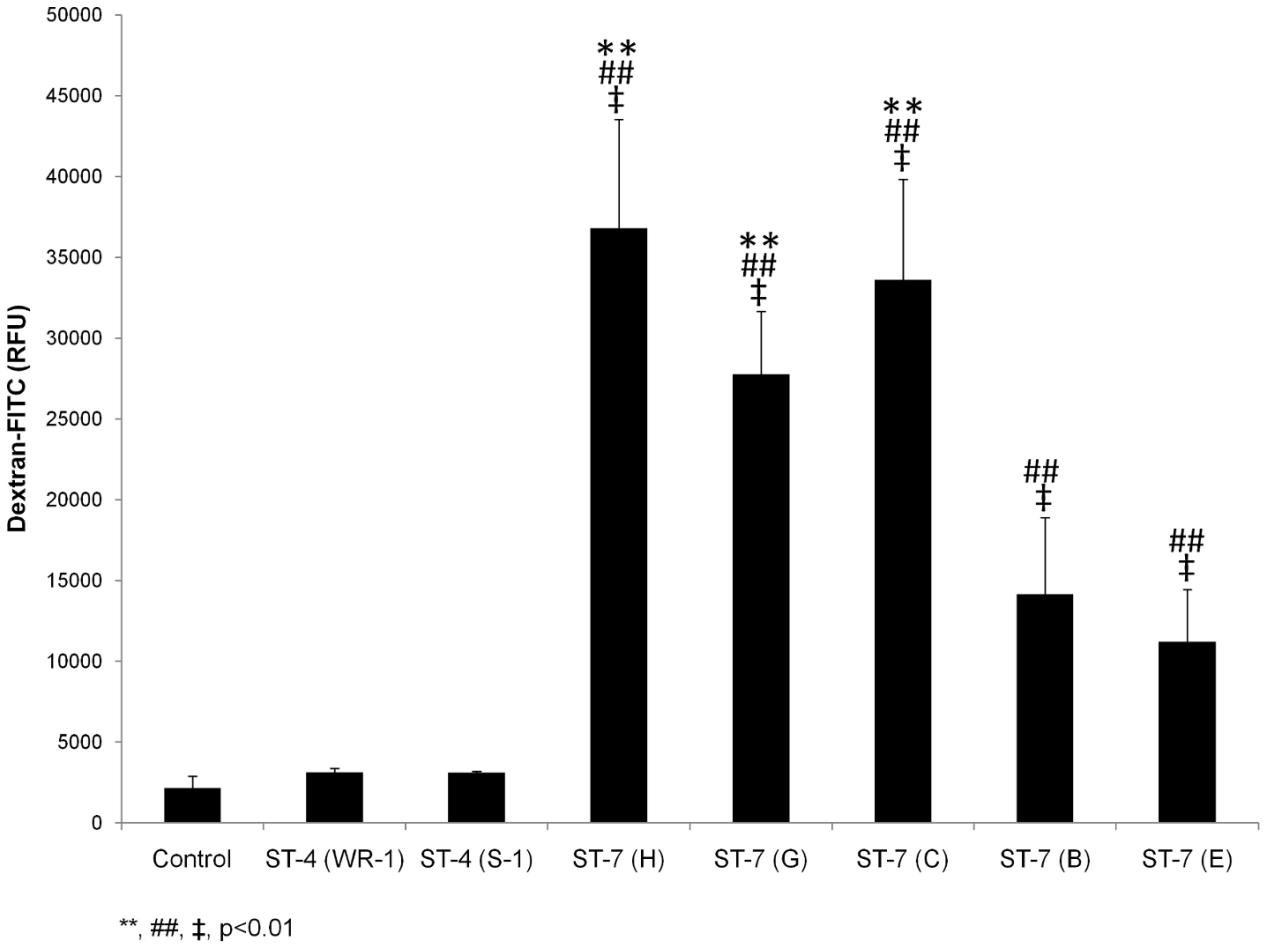
Intra-and inter-subtype variation among *Blastocystis* ST-4 and ST-7 strains in inducing permeability increase. Graph representing epithelial permeability of Caco-2 monolayers after co-incubation with live cells of *Blastocystis* ST-7 and ST-4 for 24 h. All strains in ST-7 induced significant increase in flux of dextran–FITC across epithelial monolayer of Caco-2 cells compared with control monolayers (p<0.01). No significant change in permeability was observed in *Blastocystis* ST-4-infected Caco-2.Within ST-7, an intra-subtype variation in the capability of inducing permeability change was observed. Isolates C, G and H induced much higher permeability increase compared to isolates B and E within the same subtype (p<0.01). **, p<0.01 vs. ST-7 (B, E)-infected cells; ##, p<0.01 vs. ST-4-infected cells; ‡, p<0.01 vs. non-infected cells. Each value represents mean of twelve samples, taken from three independent experiments, four samples from each. Error bars represent the standard errors.

Given that *Blastocystis* induces barrier dysfunction in an *in vitro* model [36] and that clinical and animal studies suggest an induction of barrier defects by *Blastocystis* [2], [8], [33], [62], we investigated the variability in the ability of different parasite isolates to breach the epithelial barrier. Live parasites of all *Blastocystis* isolates used were applied apically on to Caco-2 cell monolayers differentiated on transwell inserts. Permeability changes caused by the parasites were analyzed by measuring the flux of a dextran–fluorescein isothiocyanate (FITC) probe across the intestinal epithelial barrier from the apical to the basolateral compartment. Exposure to ST-4 isolates WR-1 and S-1 did not change permeability significantly compared with that of the control (Figure 1). In contrast, all five ST-7 strains of *Blastocystis* could induce significant increase in epithelial permeability compared with the control and ST-4-treated monolayers (p<0.01). Within ST-7, extensive variations in the ability to induce permeability increase were observed. Compared with the negative control monolayers, the fold increase in permeability to FITC-dextran in ST-7-infected monolayers were 16, 11.8, 14.5, 5.5, 4.2, respectively, for isolates H, G, C, B and E (Table 1). Among them, C, G and H induced a significantly higher permeability increase compared with isolates B and E (p<0.01) (Figure 1). The permeability change caused by isolate H was the most prominent and was more than three times higher than that by isolate E (Table 1). Altogether, it suggested intra- and inter-subtype variation in barrier disruptive activity of different *Blastocystis* isolates.

**Table 1:** Comparisons of effects of luminal pathogens/toxins on intestinal permeability increase.

Pathogen/Toxin	Cell Line	Strain	Infection Dose	Infection time	Permeability Increase (fold change normalized to control)	Reference
***Blastocystis***	Caco-2	ST-7 (H)	10^8^/ml	24 h	16	Current study
		ST-7 (G)			11.8	
		ST-7 (C)			14.5	
		ST-7 (B)			5.5	
		ST-7 (E)			4.2	
		ST-4 (WR-1)			NS^a^	
		ST-4 (S-1)			NS^a^	
***Clostridium difficile***	T84	*Clostridium difficile Toxin A*	10 nM	5 h	3	[106]
***Cryptosporidium parvum***	Caco-2	GCH1, UCP	2×10^5^/ml	48 h	2.14	[107]
			2×10^6^/ml		18.6	
***Entamoeba histolytica***	Human intestinal xenografts	HM1:IMSS strain	10^7^/ml	24 h	>100	[108]
						
						
***Giardia lamblia***	SCBN	NF strain	2×10^7^/ml	24 h	>4	[109]
				48 h	9	
	SCBN	S2 isolate	10^7^/ml	24 h	>40	[110]
	SCBN	S2 isolate	10^6^/ml	2 h	5.6	[111]

a NS, Not Significant.

### *Blastocystis* exhibits intra- and inter-subtype variability in ability to disrupt epithelial tight junction proteins

**Figure 2:**
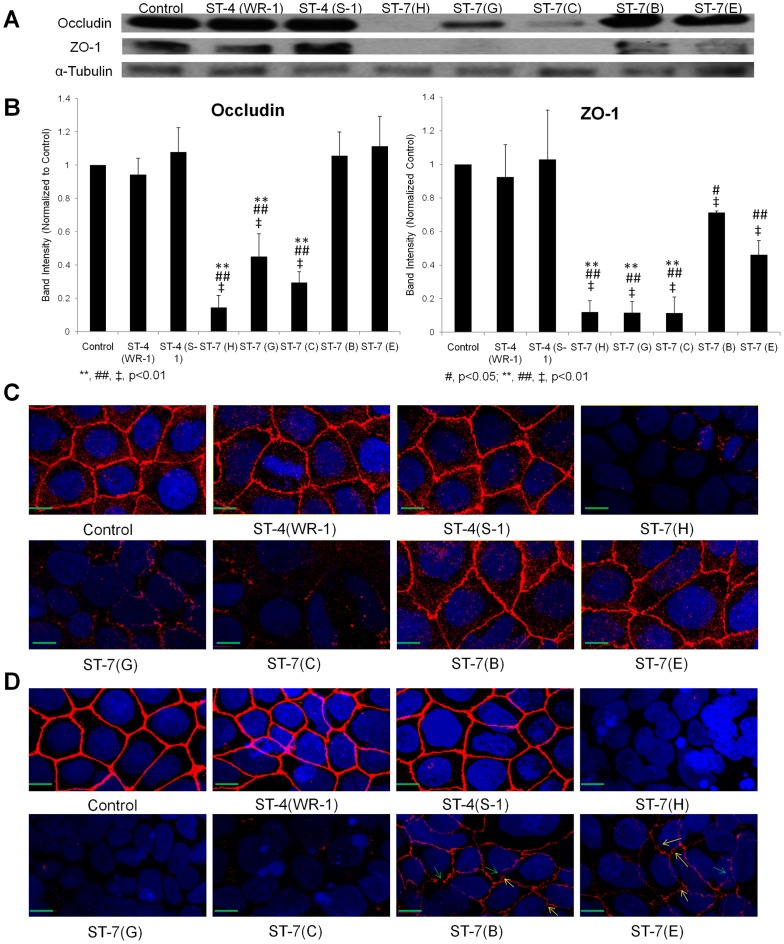
Differential effects on tight junction protein degradation in Caco-2 cell monolayers by different strains of *Blastocystis*. (A) Representative Western blot analysis of occludin and ZO-1 level in Caco-2 epithelium. Monolayers were harvested after infection with *Blastocystis* ST-4 and ST-7 isolates; normal culture media was used as the negative control. No obvious change in occludin and ZO-1 was observed in ST-4-treated samples. Within ST-7, a more prominent decrease or loss of occludin band could be seen in cells treated by isolates C, G and H, while the changes in B-, E-treated cells were not obvious; for ZO-1 tight junction protein, loss of band was observed in Caco-2 cells after coincubation with ST-7 (C, G, H), whereas a decrease in band intensity was also noticed in B- and E-treated cells. (B) Quantification of tight junction levels through densitometry analysis of Western blot radiographs. Densitometric values of occludin and ZO-1 signals were quantified and expressed as the ratio to α-Tubulin. ST-4-treated samples did not differ significantly from control in occludin and ZO-1 level; ST-7 (C, G, H) induced significant occludin and ZO-1 degradation compared with ST-4 and ST-7 (B, E) isolates (p<0.01). ST-7 (B, E), however, were able to induce significant ZO-1 degradation compared with control and ST-4, but not for occludin. **, p<0.01 vs. ST-7 (B, E)-infected cells; #, p<0.05 vs. ST-4-infected cells; ##, p<0.01 vs. ST-4-infected cells; ‡, p<0.01 vs. non-infected cells. Results were from three independent experiments. Error bars represent the standard errors. (C) Representative confocal micrographs illustrating occludin integrity in Caco-2 monolayers. Caco-2 cells are labelled with DAPI (Blue) and an antibody targeting tight junction protein occludin and Cy3® goat anti-mouse IgG (Red). Corresponding with Western blot results, in ST-4 and ST-7 (B, E)-treated samples, no obvious change in occludin was observed. Infection with ST-7 (C, G, H), however, induced focal disruptions and degradation in occludin, as shown by the decreased occludin staining intensity in Caco-2 cell line. Scale bar = 10 µm. (D) Representative confocal micrographs illustrating ZO-1 integrity in Caco-2 monolayers. Caco-2 cells are labelled with DAPI (Blue) and an antibody targeting tight junction protein ZO-1 and Cy3® goat anti-mouse IgG (Red). Compared with the negative control and ST-4-treated samples, where ZO-1 appeared continuous with sharp pericellular staining patterns, all ST-7 isolates resulted in focal disruption as well as degradation of ZO-1 in Caco-2 cells. In ST-7 (C, G, H)-treated samples, ZO-1 staining almost became invisible, indicating more degradation of the protein, which corresponded with the western blot results. Notably, treatments with ST-7 (B, E) resulted in both focal disruptions (green arrows) as well as reorganizations of ZO-1 (yellow arrows). Scale bar = 10 µm.

Tight junction (TJ) complexes regulate epithelial barrier function [63]. Given that observed previously a ST-7 isolate of *Blastocystis* induced epithelial barrier defect by disruption of tight junction protein ZO-1 [36], we proceeded to conduct an in-depth analysis of the changes in Caco-2 tight junction proteins occludin and ZO-1 after exposure to all of the seven strains of *Blastocystis*. Consistent with the permeability results, the profile of tight junction degradation shown by Western blot analysis differed from strain to strain and correlated with the degree of permeability increase caused by each strain (Figure 2A&B). ST-4 treatments did not alter the two tight junction proteins significantly which might explain the insignificant permeability increase seen above (Figure 1). Expression of occludin, a transmembrane protein which plays a direct role in the paracellular permeability [64], was significantly decreased compared with the control and ST-4 treatments by exposure to ST-7 isolates C, G, and H (p<0.01) (Figure 2A&B). But no changes in occludin were observed in ST-7 isolates B- and E-treated cells, which might contribute to the less pronounced permeability increase by B and E compared with C, G and H. The other tight junction protein ZO-1, a multi-domain scaffold protein localized at the tight junction, however, showed a different pattern of disruption from that of occludin. All ST-7 strains induced a significant decrease in ZO-1 band intensity compared with the negative control, which might explain the prominent permeability increase in B-, E-treated cells compared with control monolayers; nevertheless, the degree of ZO-1 degradation by B and E was again significantly less than C, G and H (p<0.01) (Figure 2A&B), suggesting a more potent ability in disrupting ZO-1 by C, G and H among ST-7 isolates.

In addition, immunofluorescence was performed to observe the distribution and integrity of the tight junction proteins. Indirect immunolabelling of occludin in control monolayers showed a bright and continuous band lining cell-to-cell contact regions (Figure 2C). The staining pattern in cells treated by ST-4 (WR-1 and S-1) and ST-7 (B and E) isolates did not differ from that of the controls. In contrast, infection of Caco-2 cell monolayers with ST-7 (C and G) led to focal disruptions and punctate concentration along pericellular junctions, while there was almost a complete loss of occludin in H-treated cells. As expected, ZO-1 proteins in cells after ST-4 infection appeared to be similar to those of control monolayers, where they showed a typical, continuous pericellular organization. Treatments with ST-7 (B and E), however, resulted in both a reduction in ZO-1 intensity and reorganization of ZO-1 (Figure 2D), as reported previously for ST-7 (B)-treated Caco-2 cells [36], whereas exposure to ST-7 (C, G and H) isolates led to a complete degradation of ZO-1 at the apical junctions (Figure 2D).

### *Blastocystis* isolates exhibits strain-to-strain variation in attachment to human enterocytes

**Figure 3:**
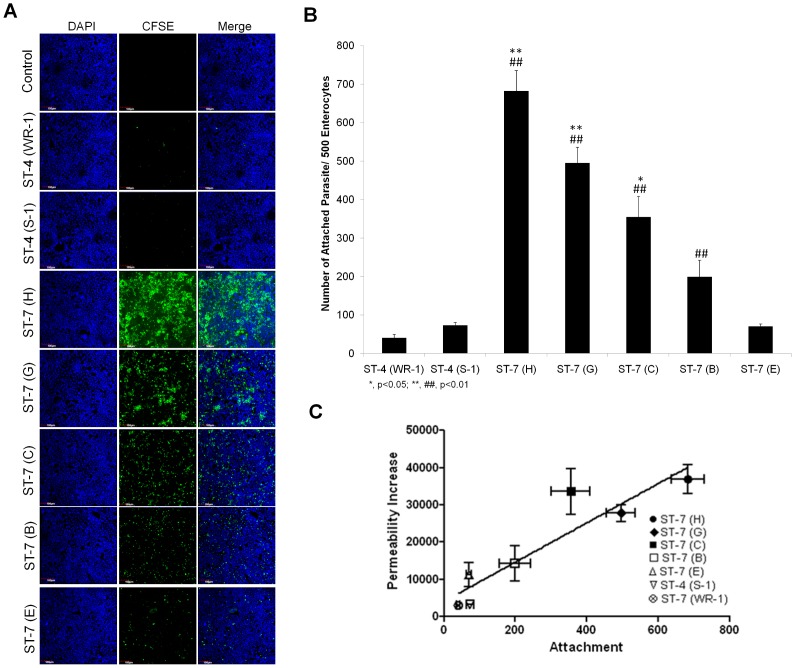
*Blastocystis* exhibits intra- and inter-subtype variation in attachment to Caco-2 cells. (A) Representative confocal micrographs illustrating intra- and inter-subtype variation in *Blastocystis* attachment to Caco-2 cells. Caco-2 monolayers were grown to confluency on glass converslips and were then co-incubated with the same number of parasites of different strains of *Blastocystis* pre-stained with CFSE (green). Normal culture media was used as a negative control. After co-incubation, the non-attached parasites were washed away. The Caco-2 monolayers were then stained with DAPI and then were viewed using confocal microscope (Olympus Fluoview FV1000; Olympus, Japan). More green in the field represents more parasites attached to the monolayer. Both ST-4 strains adhere with a negligible number. An intra-subtype variation in the number of attachment within ST-7 is obvious. Isolates C, G, H appeared to attach at a much higher level than B and E to Caco-2 cells. Scale bar = 100 µm. (B) Graph representing number of *Blastocystis* parasites attached to host cells. ST-7 strains C, G and H exhibited a significantly higher number of attached parasites than ST-4 strains and ST-7 isolates B and E. *, p<0.05 vs. ST-7 (B, E); **, p<0.01 vs. ST-7 (B, E); ##, p<0.01 vs. ST-4. Each value represents a mean of six readings derived from 3 independent experiments. Error bar represents standard error. (C) Relationship between attachment and permeability increase by *Blastocystis* ST-4 and ST-7 parasites. The data points indicate individual strains. *x* and *y* error bars indicate the standard error for the respective measurements (n = 3). The R^2^ for the trend line shown is 0.8506, and the p value is 0.0031. There is a positive correlation between the level of attachment and permeability increase (R = 0.9223).

**Figure 4:**
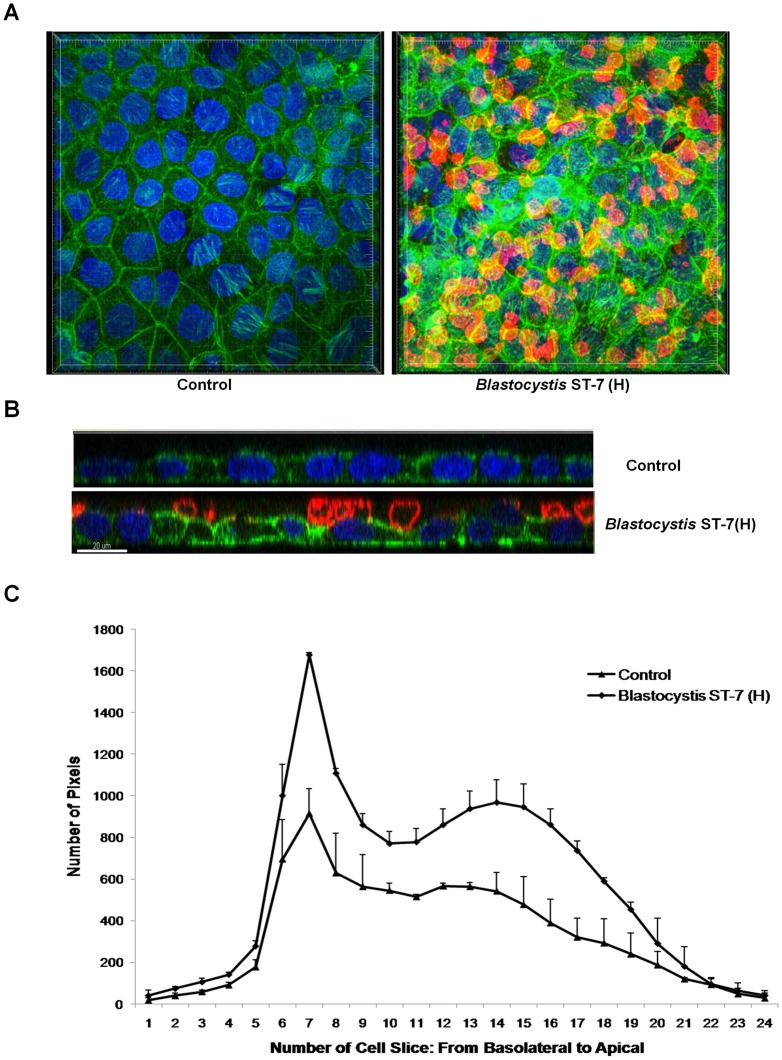
*Blastocystis* ST-7 isolate H attaches to an intestinal monolayer. Caco-2 monolayers were grown to confluency on glass coverslips and were then coincubated with *Blastocystis* ST-7 (H). Normal culture media was used as the negative control. (A) Representative confocal micrographs illustrating attachment of *Blastocystis* ST-7 (H) to Caco-2 monolayers. *Blastocystis* was labelled with legumain antibody-mAb1D5and secondary AlexaFluro 594 goat anti-mouse IgM (red). Phalloidin-FITC (green) was used to label F-actin of Caco-2 cells and DAPI (blue) for nuclei. Compared with the negative control, attachment of *Blastocystis* ST-7 to Caco-2 apical side could be seen clearly. (B) Expanded section views of epithelium of control and ST-7 (H)-treated-monolayers. Parasites could be seen intimately adhering to the epithelium and it could also be noted that the parasites adhered preferentially to the cell-cell junction site (yellow arrows) and induced an increase in actin polymerization. Compared with the negative control which displayed a properly organized epithelium, there was loss of cellular symmetry in the cells treated by ST-7 (H) (black arrowheads). Scale bar  = 20 µm. (C) Quantification of F-actin staining in *Blastocystis*-infected Caco-2 monolayers. Each cell slice (1–25) corresponds to series of images from Z-stack sections taken at 1 µm thickness through the cell monolayer. X-axis illustrates cell layers from basolateral to apical. Y-axis illustrates the number of pixels present over the entire area of image. Monolayers treated with ST-7 (H) resulted in marked increase in actin intensity compared to the negative control (p<0.01).

**Figure 5:**
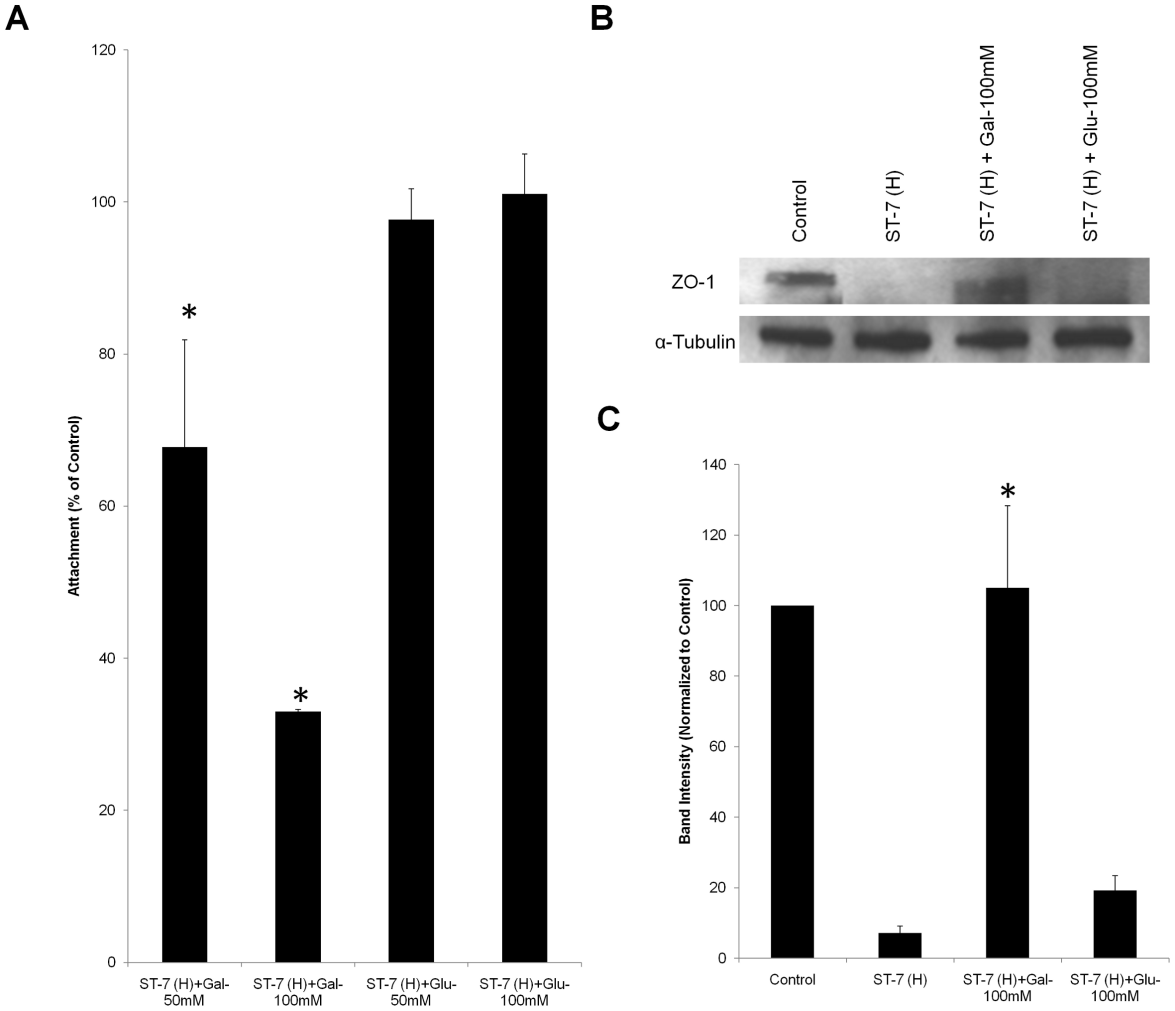
Inhibition of *Blastocystis* ST-7 (H) adhesion by galactose rescues *Blastocystis* ST-7 (H)–induced ZO-1 tight junction degradation. (A) Dose-dependent inhibition of galactose on *Blastocystis* ST-7 (H) adhesion to Caco-2 monolayers. *Blastocystis* ST-7 (H) were incubated with epithelial cells in the presence of different concentrations of galactose and glucose (50 and 100 mM, respectively). A value of 100% was assigned to number of binding parasites without addition of sugars as control. The numbers of attached parasites with galactose addition were normalized to control.*, p<0.01 vs. control. (B) Representative western blot analysis of ZO-1 level in Caco-2 epithelium. Caco-2 monolayers were infected with *Blastocystis* ST-7 (H) in the presence of saccharides galactose and glucose at 100 mM and incubated for 1 h. Monolayers were washed and prepared for western blotting. Normal culture media with no sugar addition was used as the negative control. Galactose rescued *Blastocystis*–induced ZO-1 tight junction degradation. (C) Quantification of ZO-1 levels through densitometry analysis of Western blot radiographs. Densitometric values of ZO-1 signals were quantified and expressed as the ratio to α-Tubulin. ZO-1 degradation was significantly rescued by addition of galactose. *, p<0.01 vs. ST-7 (H)-treated sample. Values are the means ± standard errors from data of three experiments. Error bars represent the standard errors. Glu, glucose; Gal, Galactose.

In many parasites and other pathogens, adhesion is directly associated with the virulence properties of the strains [40], [42]. Therefore, we tested whether there was a difference in the adhesion properties of different isolates and whether it correlated with the ability of the parasites to induce a permeability increase in intestinal monolayers. To detect and quantify adhesion of different *Blastocystis* isolates to host cells, an adhesion assay was developed using Caco-2 cell line. Both of the ST-4 strains, WR-1 and S-1, showed negligible adhesion (Figure 3A&B). Within ST-7, isolates B and E adhered to host cells in a significantly lower number than C, G and H (p<0.05) (Figure 3B). From the merged image, it was clear that C, G and H parasites attached in large numbers (Figure 3A). Isolate H, which induced highest epithelial permeability increase, was also the most adhesive to host cells (Figure 3B). The results suggested an association between the level of attachment and the ability to induce permeability increase. An analysis of these two phenotypes showed a significant positive correlation, suggesting that adherence might play a role in virulence (R^2^ = 0.8506, p<0.01) (Figure 3C). Even though isolates S-1 and E exhibited similar level of attachment (Figure 3B), the former failed to induce a permeability increase in Caco-2 monolayers (Figure 1), suggesting that additional factors of isolate E might contribute to the permeability increase. Furthermore, we also provided confocal microscopic evidence of *Blastocystis*-induced pathological changes through parasite-enterocyte contact (Figure 4). *Blastocystis* ST-7 (H) preferentially adhered at the apical junction region (Figure 4A). The percentage of parasites preferentially attached to the intercellular junctions was 80%±3.8% of all attached cells (values are means ± SE). Parasites adhered intimately with host cells at the cell-cell junction site and induced an increase in actin polymerization (Figure 4B&C). In addition, a loss of cellular symmetry in host cells was observed compared with the negative control (which displayed an intact and normally organized epithelium) (Figure 4B), indicating adhesion-mediated intestinal epithelial injury. Moreover, we tested whether disruption of adhesion could restore the pathological alterations induced by *Blastocystis*. Results obtained with the most adhesive isolate H, showed that galactose effectively decreased H adhesion in a dose-dependent manner (67.7%±14.1%, 33%±0.3% of control at 50 and 100 mM, respectively) (Figure 5A) (p<0.01 for both values), while glucose, as a control sugar, did not have any significant effect on *Blastocystis* adhesion. Importantly, the ZO-1 tight junction protein was prevented from degradation by ST-7 (H) in the presence of galactose at 100 mM (Figure 5B&C), reiterating the significant role of adhesion in mediating the pathogenesis of disease.

### *Blastocystis* exhibits extensive intra- and inter-subtype variation in cysteine protease activity

**Figure 6:**
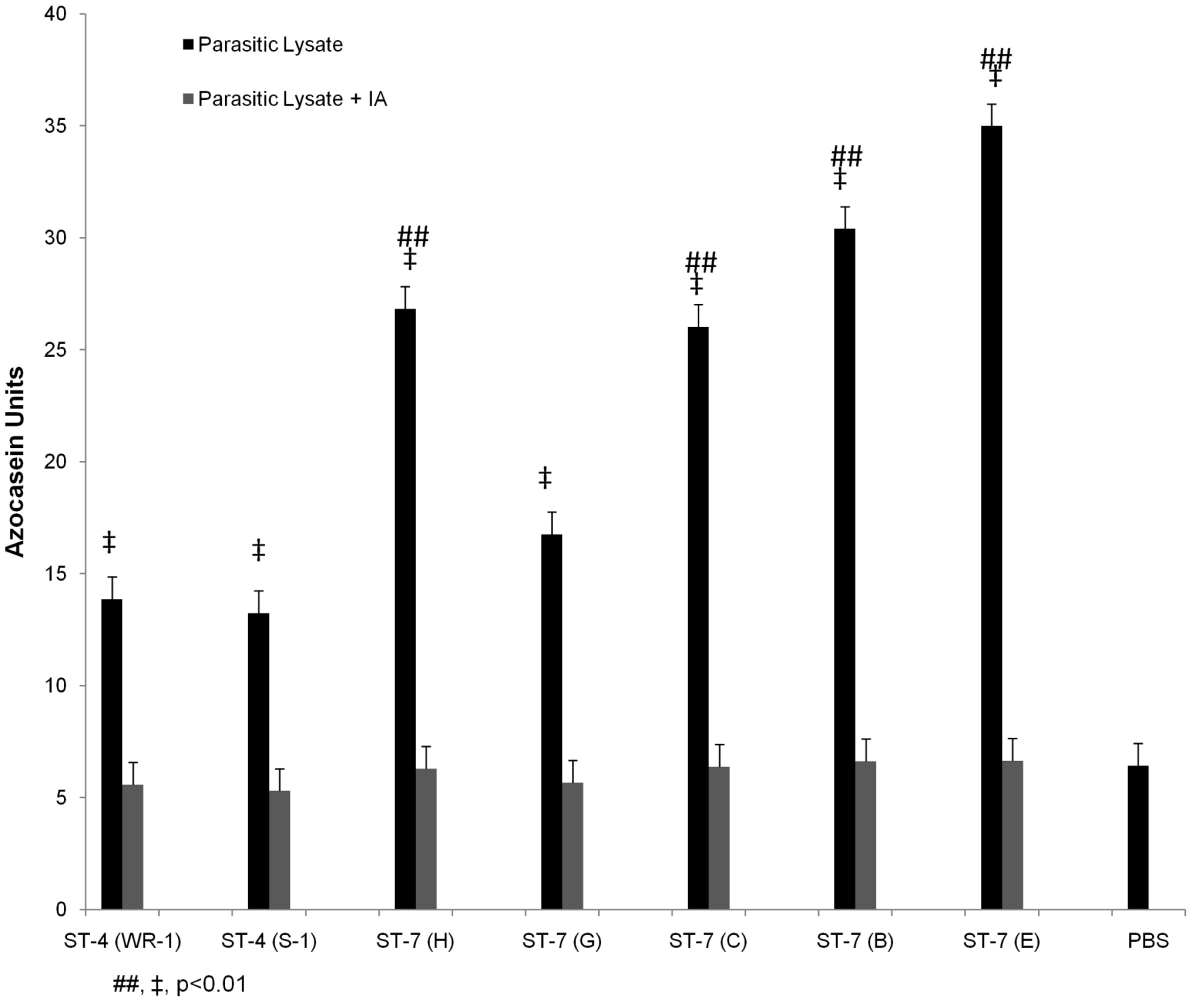
Intra- and inter-subtype variations in protease activity in *Blastocystis*. Protease activity of *Blastocystis* ST-4 and ST-7 isolates was determined by azocasein assays. Except isolate G, most of ST-7 strains exhibited significantly higher protease activities than the two ST-4 isolates (p<0.01). PBS as a background control showed activity that was significantly lower when compared to protease activities of all the isolates (p<0.01). Note that cysteine protease inhibitor iodoacetamide (IA) abolished protease activity of all the isolates, which is comparable to PBS. ##, p<0.01 vs. ST-4; ‡, p<0.01 vs. PBS control. Each value represents mean of six samples, taken from three independent experiments. Error bars represent the standard errors.

As cysteine proteases (CPs) have been suspected to be virulence factors in *Blastocystis* and shown to play a role in inducing barrier compromise [36], we compared the cysteine protease activity of all the seven *Blastocystis* isolates. CP activities in respective parasite lysates were determined using the azocasein assay. All tested strains tested showed significantly higher protease activity than the PBS control. Variation in protease activity was observed among different *Blastocystis* isolates (Figure 6). ST-4 isolates WR-1 and S-1 showed protease activity of 13.86±2.5 and 13.25±4.2 azocasein units. Except isolate G, most of the ST-7 strains showed significantly higher protease activity than ST-4 isolates (p<0.01), with 30.41±0.94, 34.99±3.04, 26.03±2.10, 26.83±4.17 azocasein units for B, E, C, and H, respectively. Lysate of G isolate showed activity of 16.77±3.98 units, which was not significantly different from ST-4 strains. There was no correlation between the total CP activities and the virulence of each strain, suggesting that total cellular cysteine protease is not responsible for the variation in pathogenicity within ST-7 isolates in inducing intestinal barrier dysfunction.

### *Blastocystis* ST-7 isolates exhibit extensive variation in resistance to Mz, and the level of Mz resistance is inversely correlated with an ability to attach and induce a permeability increase

**Figure 7:**
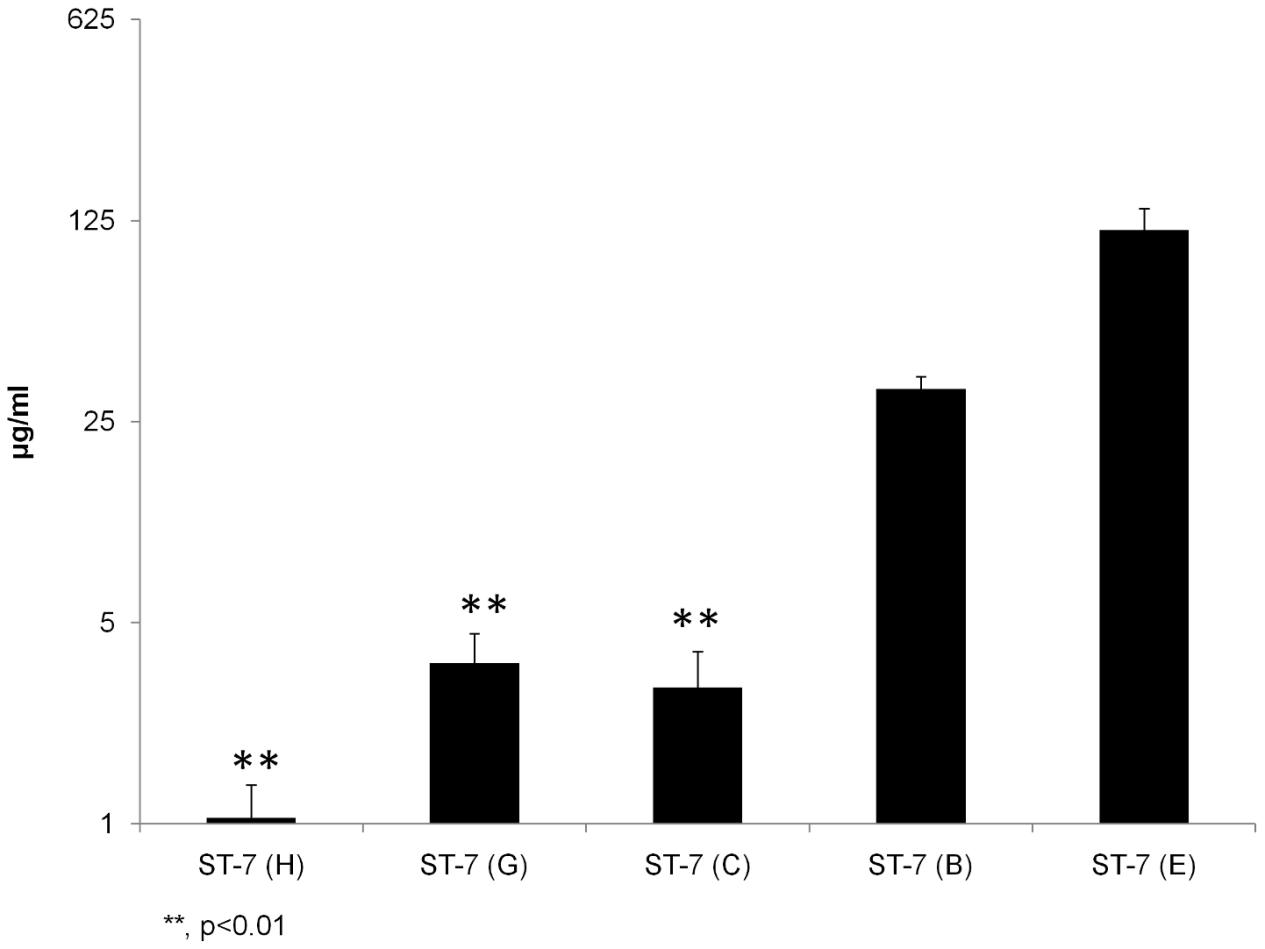
*Blastocystis* exhibits intra-subtype variation in susceptibility and resistance to Mz. Graph representing IC50s of Mz against *Blastocystis* ST-4 and ST-7 isolates tested in the study using the resazurin assay. Y axis was presented on a logarithmic scale in base 5. The IC50s of Mz against ST-7 isolates C, G and H were found to be significantly lower than those of isolates B, E within the same subtype (p<0.01). **, p<0.01 vs. ST-7 (B, E). Each point represents a mean of nine readings derived from three independent experiments, triplicate each. Error bars represent the standard errors.

**Figure 8:**
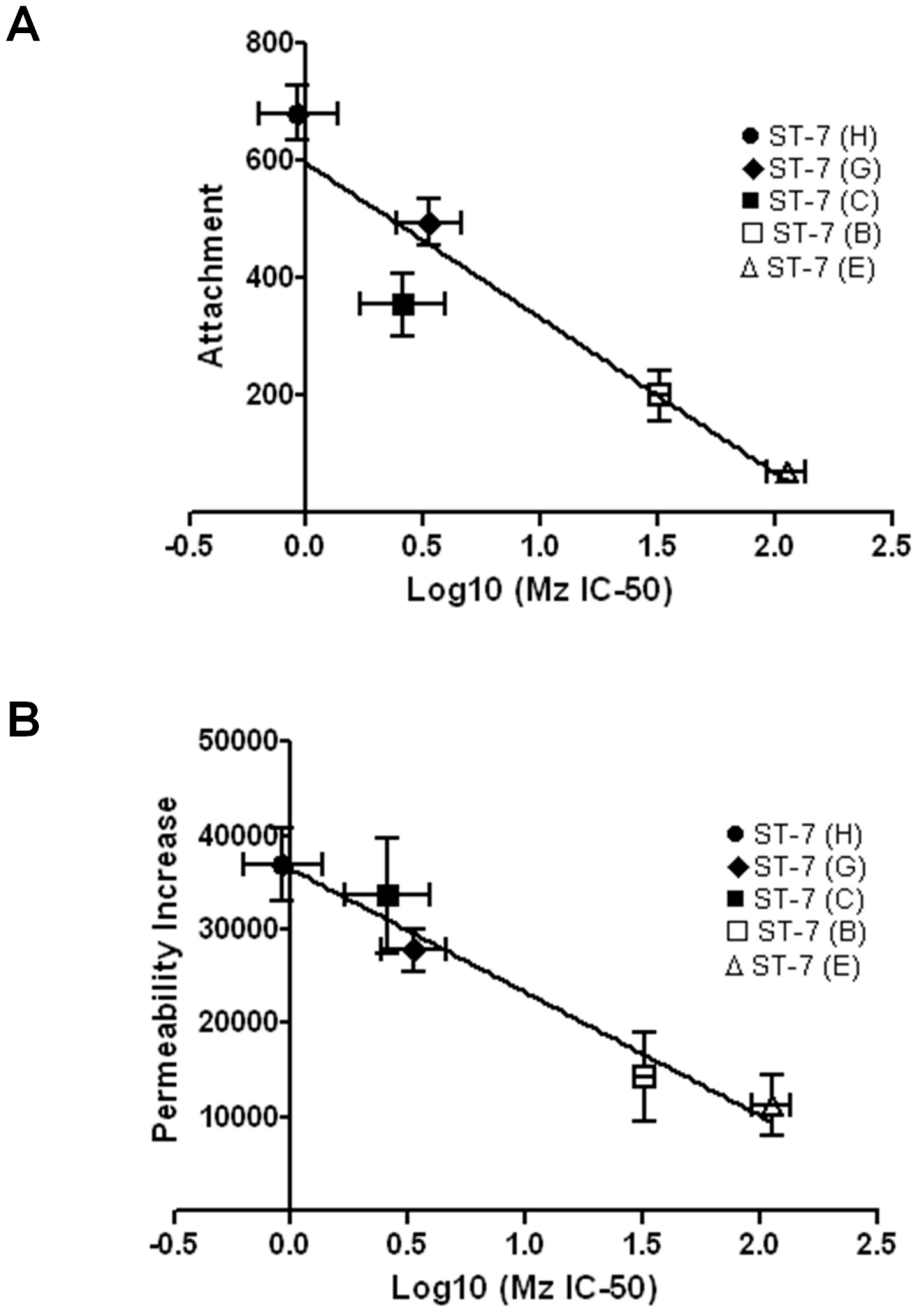
Correlation analyses between Mz resistance and (A) attachment, as well as (B) permeability increase in *Blastocystis* ST-7 parasites. (A) Relationship between Mz resistance and attachment for *Blastocystis* ST-7 parasites. The data points indicate individual strains. Error bars indicate the standard error for the respective measurements (n = 3). There was a negative correlation between the level of resistance and attachment (p<0.05, R^2^ = 0.8908). (B) Relationship between Mz resistance of *Blastocystis* ST-7 parasites and their ability in inducing permeability increase. The data points indicate individual strains. Error bars indicate standard errors for the respective measurements (n = 3). There was a negative correlation between the level of resistance and permeability increase (p<0.01, R^2^ = 0.9644).

Previously, we reported that isolates B and E belonging to ST-7 were Mz resistant [49]. As both of them also exhibited impaired ability to adhere to host cells, we tested whether there was an association between attachment and Mz resistance in ST-7, as seen in *Giardia* [54]. IC-50s of C, G and H isolates (2.98±0.97, 3.63±0.96, 1.05±0.32 µg/ml, respectively; Table 2) were significantly lower than the previously reported IC50s of isolates B and E (p<0.01; Figure 7), indicating an intra-subtype variation in Mz resistance in *Blastocystis* ST-7 parasites. Correlation analysis showed a significant negative correlation between the level of Mz resistance of an isolate and its ability to adhere to host cells (R^2^ = 0.8908, p = 0.0158) (Figure 8A and Table 3). Our results suggest that Mz resistance might entail an attachment defect in *Blastocystis* ST-7 strains.

**Table 2:** IC50s of Mz and nitric oxide donors against *Blastocystis* ST-7 isolates (µg/ml).

	ST-4		ST-7				
	Mz-susceptible					Mz-resistant	
	WR-1	S-1	H	G	C	B	E
Mz	5.5±2.89^a^	0.75±0.04^a^	1.05±0.32	3.63±0.96	2.98±0.97	32.5±3.4^a^	115.6±21.6
GSNO	83.3±2.5^b^	98.29±0.73	121.73±8.2	75.24±3.99	61.79±4.03	30.63±1.67^b^	30.92±1.74
NaNO_2_	233±9.2	240.6±10.4	217.45±16	162.15±34	199.75±5.8	86.47±12.8	106.35±4.8

a See Reference[49].

b See Reference[30].

**Table 3:** Correlation of Mz resistance with attachment, permeability increase and nitric oxide IC-50 in *Blastocystis* ST-7.

	Pearson Correlation (R)	R2	P value
**Attachment**	−0.9438	0.8908	0.0158^a^
**Permeability Increase**	−0.9821	0.9644	0.0029^a^
**Nitric Oxide IC-50**	−0.8929	0.7972	0.0414^a^

a Correlation is significant at the 0.05 level (2-tailed).

As we showed that the level of attachment was correlated with the ability to induce permeability increase (Figure 3C), the impaired attachment in Mz^r^ strains might indicate a less potent ability in inducing a permeability increase. Indeed, the correlation analysis between drug resistance and permeability increase showed a significant negative correlation (R^2^ = 0.9644, p<0.01) (Figure 8B and Table 3). Our results suggested that Mz resistance was negatively correlated with barrier disruptive ability of *Blastocystis* which in turn dampens its virulence.

### Mz resistance in *Blastocystis* does not lead to decrease in cell proliferation in *Blastocystis* ST-7

Most drug resistance mechanisms are associated with a fitness cost that is typically observed as a reduced growth rate [51], [65]. To investigate the functional consequences of Mz resistance in *Blastocystis*, we examined the proliferative potential of Mz^r^ and Mz^s^ strains from growth curves assayed over 96 h. The growth rates of Mz^r^ isolates were not always lower than Mz^s^ isolates (Figure S2). Isolate E, being highly resistant to Mz, consistently grew more rapidly than Mz^s^ isolates C and G, suggesting that drug resistance in *Blastocystis* ST-7 might not necessarily result in slower growth.

### Extensive variation in resistance to nitrosative stress among *Blastocystis* ST-7 isolates

Aside from adhesion, microorganisms also need to evolve means to counter the host immune system for effective colonization in the intestinal lumen [37]. Nitric oxide (NO) is a potent innate immune response against a range of pathogens and nitrosative stress prevents colonization of host tissues by parasites and bacteria susceptible to NO [66], [67]. Previous studies [30], [68] have already suggested NO induces cell death in *Blastocystis*. Thus, the ability of *Blastocystis* to tolerate nitrosative stress is likely to be important for its survival in the gut lumen. Previously, we observed that a Mz^r^ strain of *Blastocystis* exhibited lower tolerance to nitric oxide toxicity compared with a Mz^s^ strain [30]. Here, we tested the IC50s of NO donors against the Mz^r^ and Mz^s^ strains, to examine their ability to tolerate nitrosative stress. Interestingly, Mz^s^ isolates C, G and H of *Blastocystis* ST-7, all exhibited significantly higher IC-50s of GSNO (61.79±4.03, 75.24±3.99, 121.73±8.17 µg/ml, respectively; p<0.01; Figure S3A and Table 2) than Mz^r^ isolates B and E (30.63±1.67, 30.92±1.73 µg/ml, respectively). Therefore, Mz^r^ strains were more susceptible to GSNO. An analysis again showed a significant negative correlation between the level of Mz resistance of an isolate and its ability to tolerate GSNO toxicity (R^2^ = 0.7972, p<0.05) (Figure S3B and Table 3). IC50s with another NO donor NaNO_2_ for all these strains exhibited similar trends to those with GSNO (Table 2). Taken together, the results suggested that, although Mz resistance helps *Blastocystis* survive stress from chemotherapy, it makes Mz^r^ strains less capable of coping with nitrosative stress.

Concomitantly, to determine the morphological changes resulting from nitrosative stress, isolates H and B were stained with propidium iodide and annexin V-FITC after exposure to NO [35], [49]. Necrotic cells incorporated both PI and annexin V-FITC stain and cells undergoing programmed cell death bind annexin V-FITC alone. After treatment with 50 µg/ml concentration of GSNO, features of both necrosis and apoptosis were observed in the Mz^r^ isolate B, whereas the Mz^s^ isolate H were less affected (Figure S3C). Compared with cultures of C, G and H, Mz^r^ isolate B and E exhibited a significantly higher percentage of cells undergoing cell death (37.54% and 32.93% for B and E respectively; 12.38%, 10.2%, 7.21% for isolates C, G and H, respectively; p<0.01) (Figure S3D), which also indicates that the parasite cell death induced by NO was predominantly by necrosis with a minority of cells dying by apoptosis.

## Discussion

Although it is one of the commonest eukaryotic organisms present in the alimentary tract of human and nonhuman hosts worldwide [69], the debate about the pathogenicity of *Blastocystis* continues [3], [21], [70]–[72], and arguments for and against its pathogenicity both abound [69]. The enigmatic role of *Blastocystis* has mainly been due to a lack of basic knowledge about its biology and convincing evidence of its pathogenicity [69]. A major obstacle or challenge is the potential for intra- and inter-subtype variation in *Blastocystis* pathogenicity [1], [21]. Although previous research attempted to validate this argument by using animal models [32], the number of strains used were limited and no consensus on the appropriate *in vivo* infection model was reached for *Blastocystis*; thus, the infection outcomes might be reflective of infectivity rather than pathogenicity. Our study, using a well-established *in vitro* system mimicking the host-pathogen interplay, provides, for the first time, a comprehensive analysis of different strains from two clinically relevant subtypes, and gives evidence that *Blastocystis* exhibited not only inter- but also intra-subtype variability in causing barrier dysfunction. Disturbances in epithelial barrier function are commonly associated with intestinal inflammatory disorders and have been reported in symptomatic cases of *Blastocystis* infection [2], [4], [62], [73]. Thus, our findings might help explain the conflicting data concerning the inconsistency in reports of *Blastocystis*-induced intestinal inflammatory disorders. Besides, compared to other pathogens, *Blastocystis* requires a higher infectious dose to induce comparable damage (Table 1). Previous studies have also shown that greater *Blastocystis* parasite load shows higher pathogenicity [15], [17]. Notwithstanding, some *Blastocystis* isolates (e. g., C and H) could induce intestinal barrier compromise at a level comparable to accepted pathogens like some of the *Giardia* and *Cryptosporidium* strains (Table 1).

Paracellular permeability was regulated by tight junctions, which seal the space between neighboring cells, generating an impermeable barrier between the epithelium and the extracellular environment, protecting deeper tissues from external aggressions including microbial infections [74]. However, this first line of defense against infection has become one of the most exploited gates for pathogens to access and colonize the host organism [74]. Enteric pathogens have developed a broad and complex range of mechanisms to subvert the host tight junction [75]. In general, key mechanisms identified to date include direct rearrangement or degradation of specific tight junction proteins, reorganization of the cell cytoskeleton, and activation of host cell signaling events [76]–[78]. In this study, we observed disruption of tight junctions (TJs) which mainly resulted from degradation in at least two TJ proteins: ZO-1 protein, which is linked to the cytoskeleton and plays a pivotal role in the TJ architecture [79], and occludin, which is important in maintaining the integrity and barrier function [64], [80]. Previously, we reported that *Blastocystis* phophorylates MLC and leads to cytoskeletal and ZO-1 rearrangement [36]. Indeed, we also observed myosin light chain phosphorylation and TJ rearrangement in B- and E-treated Caco-2 cells besides degradation of ZO-1 protein (Figure S4). However, for *Blastocystis* isolates C, G, and H, although MLC was phosphorylated, degradation of TJs appeared to be the main mechanism, indicating the strategies utilized by *Blastocystis* to induce permeability increase are likely to be diverse and different isolates may have developed different major mechanism to compromise barrier function. In our previous study, the observation that ROCK inhibition did not completely rescue the increase in the epithelial permeability induced by the parasite [36] also suggested that *Blastocystis* utilizes more than one mechanism to breach the epithelial barrier.

Epithelial attachment is an important factor determining the persistence and virulence of luminal pathogens [38], [39]. Indeed, in our study, pathogenicity of *Blastocystis* was also found to be correlated with their ability to attach to epithelial cells. Importantly, inhibition of adhesion ameliorates the parasite's pathogenic effects on degradation of ZO-1 tight junction protein. How adhesion leads to intestinal barrier dysfunction needs to be investigated further. *Giardia intestinalis* was shown to produce harmful substances as a result of the host-pathogen contact [81]. Several enzymes from the secreted products have been identified in *G. intestinalis*, which are suggested to facilitate effective parasite adhesion and colonization of the human small intestine [82], [83]. Whether the adhesion of *Blastocystis* with host cells also enables particular enzymes to be released and to participate in pathogenesis would be interesting to investigate. The *Blastocystis* genome has been available and *in silico* analysis of the ST-7 secretome predicted 75 putative secreted proteins, some of which may have a direct connection with pathogenicity [34]. Recently, two cysteine proteases (legumain and cathepsin B) have been characterized in the ST-7 culture supernatant; these enzymes showed proteolytic activities by gelatin zymograms [84]. However, whether these secreted proteins act on intestinal cells and disturb gut function has not been studied. It would be interesting to investigate whether adhesion of *Blastocystis* to the epithelium might trigger the parasite to actively produce virulence factors that possibly activate signaling cascade leading to tight junction disruption. The observation of preferential adherence of cytopathic *Blastocystis* strains to intercellular junctions also raised the interesting question as to whether the preferred attachment at junctional area facilitates tight junction degradation by *Blastocystis*.

Parasites such as *Giardia intestinalis* and *Entamoeba histolytica* have distinct virulent and non-virulent strains that may be attributable to qualitative and quantitative variation in their cysteine proteases activity [60], [85], [86]. *Blastocystis* cysteine proteases are also implicated in the activation of NF-κB in colonic epithelium, leading to an upregulation of the pro-inflammatory cytokine IL-8 [87]. Our most recent study [36] has also shown that cysteine proteases could cause human epithelial barrier compromise. Indeed, isolate E, even though being the least adhesive in ST-7, has yet the highest cysteine protease activity, which might explain the permeability increase observed in E-infected epithelium; while isolate S-1, with equivalent level of attachment to E, and at the same time the lowest CP activity, failed to induce any increase in intestinal permeability. Interestingly, the highly adhesive G isolate, though with similar CP activity to ST-4 isolates, could induce prominent epithelial barrier disruption, highlighting the important role of adhesion in mediating intestinal pathology. Taken together, both adhesion and CP activity might contribute to *Blastocystis*–induced barrier dysfunction. ST-4 isolates, low in both adhesion and CP activity, appeared to be avirulent for human intestinal monolayer, whereas all ST-7 isolates studied are capable of infection because of being either adhesive or exhibiting high CP activity, or both. Moreover, adhesion is a major determinant in the pathogenicity of a strain. The results of our study provide evidence that the pathogenesis of blastocystosis is complex and parasite adhesion and cysteine proteinases might be important virulence factors. Specific virulence factors and their mechanisms remain to be explored in depth.

Besides the observation that *Blastocystis* ST-7 exhibited intra-subtype variation in epithelial attachment, we also observed an association between epithelial attachment and Mz susceptibility. Mz^r^ isolates in ST-7 exhibited a defect in epithelial attachment. How Mz resistance would affect *Blastocystis* adhesion to host cells remains to be addressed. Drug resistance has been shown to modulate adhesion in some bacterial and parasitic agents, such as *Escherichia coli, Giardia*, and *Stenotrophomonas* [54], [88]–[94]. Tejman-Yarden *et al*. [54] showed that in *Giardia lamblia*, epithelial attachment was linked to glucose metabolism. Glucose promoted parasite attachment and Mz^r^ lines were shown to consume less glucose than their parental Mz^s^ lines, providing a possible explanation for the attachment defect observed in Mz^r^ lines. In *Blastocystis*, we also measured the glucose consumption of all seven strains used in the study. However, their respective glucose consumption rate did not correlate with the level of Mz resistance (Figure S5), suggesting that glucose metabolism may play a different role in *Blastocystis* from that in *Giardia*. In addition, it was suggested that specific variable surface proteins (VSPs) might play a role in *Giardia* adherence and that to analyze the impact of Mz resistance on the VSP pattern may reveal new insights into the functions of specific VSPs in parasite attachment [54]. In *Blastocystis*, whether the decreased adhesion observed in Mz^r^ strains indicated that Mz selection negatively affected the synthesis or expression of surface adhesion molecules might be worth exploring. On the other hand, little is known about the nature of adhesion of *Blastocystis* to host cells and many basic questions (such as what molecules mediate parasite-host adhesion and what factors influence the process) need to be addressed. Further dissection of the mechanisms of *Blastocystis* attachment to host cells will shed new light on the association between Mz resistance and attachment.

Mz^r^ strains not only exhibited a defect in attachment, they also showed impaired NO tolerance in *Blastocystis* ST-7. NO is an important aspect in human innate immunity and limits microbial persistence in the gut by inducing cell death and preventing their growth and encystations [66]. Therefore, the ability to tolerate nitrosative stress would be an important aspect for parasite survival. Our study suggests that Mz resistance might limit its survivability to host innate immune response, such as coping with nitrosative stress. How Mz resistance affects the susceptibility to NO needs to be investigated further. Development of Mz resistance in microorganisms is a multifactorial process and the mechanisms are diverse [95]. In *Giardia* and *Trichomonas*, Mz resistance usually develops due to impairment of Mz activation enzymes in the parasite. For example, resistance to Mz has been associated with down-regulation or decreased activity of pyruvate: ferredoxinoxidoreductase (PFOR), which activates the 5-nitroimidazole (5-NI) pro-drugs to the toxic radical states inside the parasite in conjunction with the electron acceptor ferredoxin [54], [96], [97]. These Mz activation enzymes are oxygen-sensitive and are also important components of parasite anaerobic respiratory system. Anaerobic respiratory enzymes also play an essential role in free radical (e.g., H_2_O_2_ or NO) scavenging and, if these enzymes are impaired, as expected in Mz resistant parasite strains, it would lead to decreased free radical tolerance in these isolates [98], [99]. However, the mechanism of Mz activation has not been studied in *Blastocystis*, although PFOR and other oxidoreductase enzymes are present in the organism [34], [100]. Further investigation of Mz activation and/or resistance mechanisms as well as NO-scavenging mechanisms in *Blastocystis* would be necessary to elucidate the association between NO susceptibility and Mz resistance. Recent studies of *Giardia* and *Trichomonas* suggested that Mz was activated by the nitroreductase activity of flavin-dependent thioredoxinreductase and Mz resistance was associated with decreased NADPH oxidase activity [95], [101]. A flavohemoglobin (FlavoHb), which plays a pivotal role in NO detoxification, has been characterized recently in *Giardia intestinalis* [102]. Interestingly, other studies have suggested flavoHb also demonstrates NADPH oxidase activity [103]. Furthermore, flavoHb seems to be important in the pathogenesis of disease [104], [105]. It would be intriguing to explore whether flavoHb or its homolog exists in *Blastocystis* and whether it plays any function in Mz resistance, NO-scavenging or pathogenesis.

In conclusion, our study, for the first time, showed extensive intra- and inter- subtype variability in pathogenicity in *Blastocystis* ST-4 and ST-7 *in vitro*. The existence of both pathogenic and apathogenic isolates might help explain the disparity in symptoms of among patients with *Blastocystis* infection. The modes of pathogenesis of blastocystosis are diverse, and the range of clinical symptoms observed is unlikely to be attributed to a single virulence factor or pathogenic mechanism. Moreover, our study indicates that adhesion is an important biomarker for the pathogenicity of the parasite and subsequent outcome of infection/disease, and that adhesion can be impaired by Mz resistance. Interestingly, the five ST-7 strains tested here were morphologically indistinguishable from each other, but varied significantly in many aspects of their pathobiology, including attachment and drug resistance. A deeper analysis and systematic comparison of the genome and protein expression profiles among these *Blastocystis* strains could aid in the identification and analysis of the key factors associated with the pathobiology of blastocystosis.

## Supporting Information

Figure S1 *Blastocystis* ST-4 and ST-7 strains used in the study. (A) Phylogenic tree of *Blastocystis* ST-4 and ST-7 strains based on the small subunit ribosomal RNA gene sequences. The evolutionary history was inferred using the Neighbour-Joining method. The tree was drawn to scale, with branch lengths in the same units as those of the evolutionary distances used to infer the phylogenetic tree. The evolutionary distances were computed using the Maximum Composite Likelihood method and are in the units of the number of base substitutions per site. The analysis involved 8 nucleotide sequences which are available in GenBank [55]. The GI numbers are 51291182, 51291156, 51291129, 51291116, 51291092, 51291080 and 51291104, respectively, for isolates WR-1, S-1, H, G, C, B and E. *Proteromonas lacerate* was used as the outgroup with the GI number 1304397. The tree clearly clusters strains belong to one subtype together. (B) Micrographs representing morphology of the vacuolar forms of *Blastocystis* ST-4 and ST-7 strains in axenic laboratory cultures. Scale bar = 20 µm.
(TIF)

Figure S2 Mz resistance in *Blastocystis* does not lead to decreased growth in *Blastocystis* ST-7. Graph representing growth curve of *Blastocystis* ST-7 isolates tested in the study over a period of 96 h. The growth rates of Mz^r^ isolates B and E were not significantly different from that of Mz^s^ isolates C, G and H within the same subtype. Each point represents a mean of 6 readings derived from two independent experiments, triplicate each. Error bars represent the standard errors.
(TIF)

Figure S3 Mz resistant isolates in *Blastocystis* ST-7 exhibit susceptibility to nitric oxide. (A) Graph representing IC50s of NO donor GSNO against *Blastocystis* ST-7 strains tested. ST-7 isolates C, G and H showed significantly higher tolerance to GSNO (p<0.01) than Mz^r^ strains B and E. **, p<0.01 vs. ST-7 (B, E). Each point represents a mean of nine readings derived from three independent experiments, triplicate each. The error bar represents standard errors. (B) Relationship between Mz resistance and GSNO resistance in *Blastocystis* ST-7 parasites. The data points indicate individual strains. Error bars indicate the standard error for the respective measurements (n = 3). There was a negative correlation between level of Mz resistance and tolerance of nitric oxide toxicity (p<0.05, R^2^ = 0.7972). (C) Confocal micrographs illustrating cell death features of *Blastocystis* isolates H and B under nitrosative stress. *Blastocystis* were cultured under culture conditions, in the presence of 50 µg/ml of GSNO. The cells were then stained with Annexin-V-FITC and PI to identify cells undergoing cell death. Features of necrosis (yellow arrows) and programmed cell death (red arrows) were observed. *Blastocystis* cells undergoing necrosis incorporated both PI and annexin V-FITC stain, cells undergoing programmed cell death, on the other hand bind annexin V-FITC alone. A significantly higher number of dying cells were observed in cultures of ST-7 (B) compared with ST-7 (H) cultures. Scale bar = 10 µm. The micrographs are representative of 15 pictures taken in three separate experiments (five from each). (D) Graph representing percentage of cell death in *Blastocystis* ST-7 strains after treatment with NO donor GSNO. Compared with Mz^s^ isolates ST-7 (C, G, H), percentages of dead cells in ST-7 (B, E) are significantly higher (p<0.01). **, p<0.01 vs. ST-7 (B, E). Each value represents a mean of nine readings derived from three independent experiments, triplicate each. Error bars represent the standard errors.
(TIF)

Figure S4 Differential effects on myosin light chain phosphorylation in Caco-2 cell monolayers by different strains of *Blastocystis*. (A) Representative Western blots exhibiting level of MLCp after incubation with *Blastocystis* ST-4 and ST-7 parasites. Total MLC was used as a loading control. An obvious increase in MLCp could be noticed in ST-7-treated samples. (B) Graph represents MLCp/MLC ratio normalized to the negative control in Caco-2 cells calculated through densitometry analysis of Western blot radiographs. A significant increase in MLCp was observed in all ST-7-treated Caco-2 epithelium compared with the negative control and ST-4-treated samples (p<0.01). ‡, p<0.01 vs. control. Error bars represent the standard error of the mean of 3 values obtained from three independent Western blots.
(TIF)

Figure S5 Differential consumption rate of glucose by *Blastocystis* ST-4 and ST-7 strains. Different strains of *Blastocystis* from ST-4 and ST-7 were cultured in medium containing 5 mM glucose and incubated under anaerobic conditions, and levels of glucose remaining in the medium were measured after a 24-h incubation period. Glucose consumption was expressed as the mean glucose consumption for 10^6^ cells over the period (mmol/day). No correlation was found between the rate of glucose consumption and the level of attachment or Mz resistance. Each value represents a mean of nine readings derived from three independent experiments, triplicate each. Error bars represent the standard errors.
(TIF)
