# Supplementary figures and images for: Intra-Subtype Variation in Enteroadhesion Accounts for Differences in Epithelial Barrier Disruption and Is Associated with Metronidazole Resistance in Blastocystis Subtype-7
Source: PLoS Negl Trop Dis. 2014 May 22;8(5):e2885. doi: 10.1371/journal.pntd.0002885 (PMC4031124; doi:10.1371/journal.pntd.0002885)

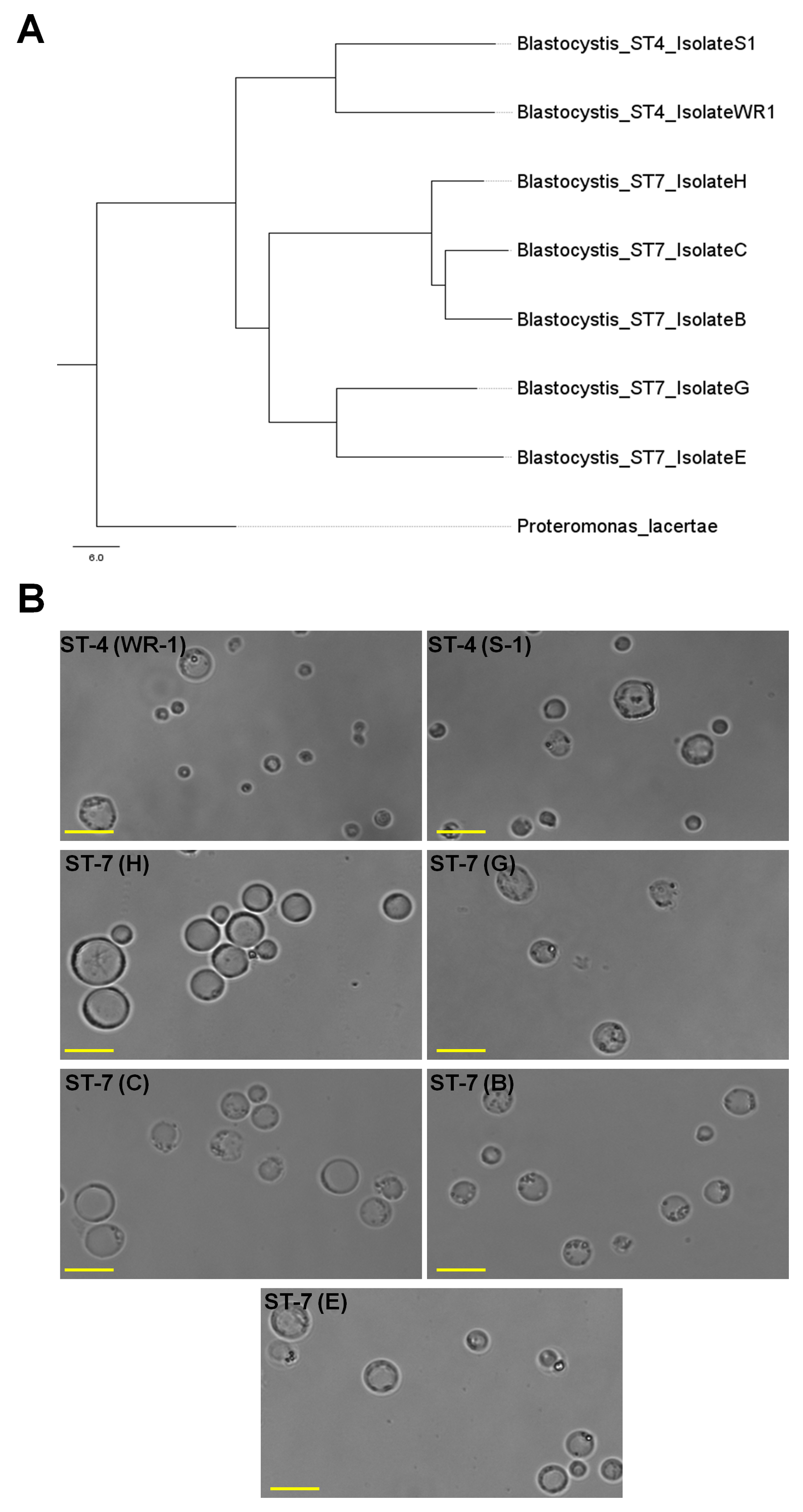

Supplement: Figure S1 — Blastocystis ST-4 and ST-7 strains used in the study. (A) Phylogenic tree of Blastocystis ST-4 and ST-7 strains based on the small subunit ribosomal RNA gene sequences. The evolutionary history was inferred using the Neighbour-Joining method. The tree was drawn to scale, with branch lengths in the same units as those of the evolutionary distances used to infer the phylogenetic tree. The evolutionary distances were computed using the Maximum Composite Likelihood method and are in the units of the number of base substitutions per site. The analysis involved 8 nucleotide sequences which are available in GenBank [55]. The GI numbers are 51291182, 51291156, 51291129, 51291116, 51291092, 51291080 and 51291104, respectively, for isolates WR-1, S-1, H, G, C, B and E. Proteromonas lacerate was used as the outgroup with the GI number 1304397. The tree clearly clusters strains belong to one subtype together. (B) Micrographs representing morphology of the vacuolar forms of Blastocystis ST-4 and ST-7 strains in axenic laboratory cultures. Scale bar = 20 µm. (TIF) [file pntd.0002885.s001.tif]

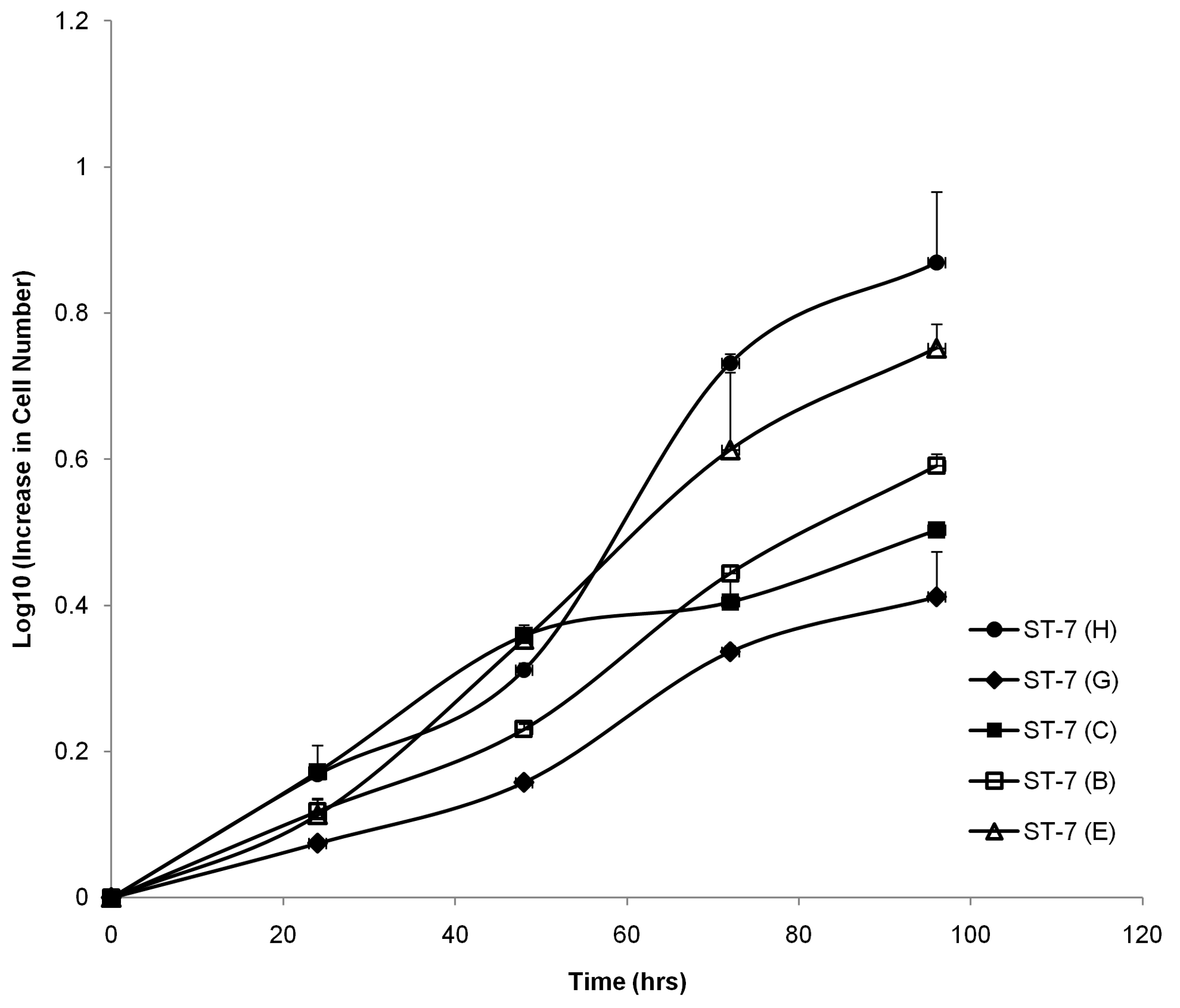

Supplement: Figure S2 — Mz resistance in Blastocystis does not lead to decreased growth in Blastocystis ST-7. Graph representing growth curve of Blastocystis ST-7 isolates tested in the study over a period of 96 h. The growth rates of Mzr isolates B and E were not significantly different from that of Mzs isolates C, G and H within the same subtype. Each point represents a mean of 6 readings derived from two independent experiments, triplicate each. Error bars represent the standard errors. (TIF) [file pntd.0002885.s002.tif]

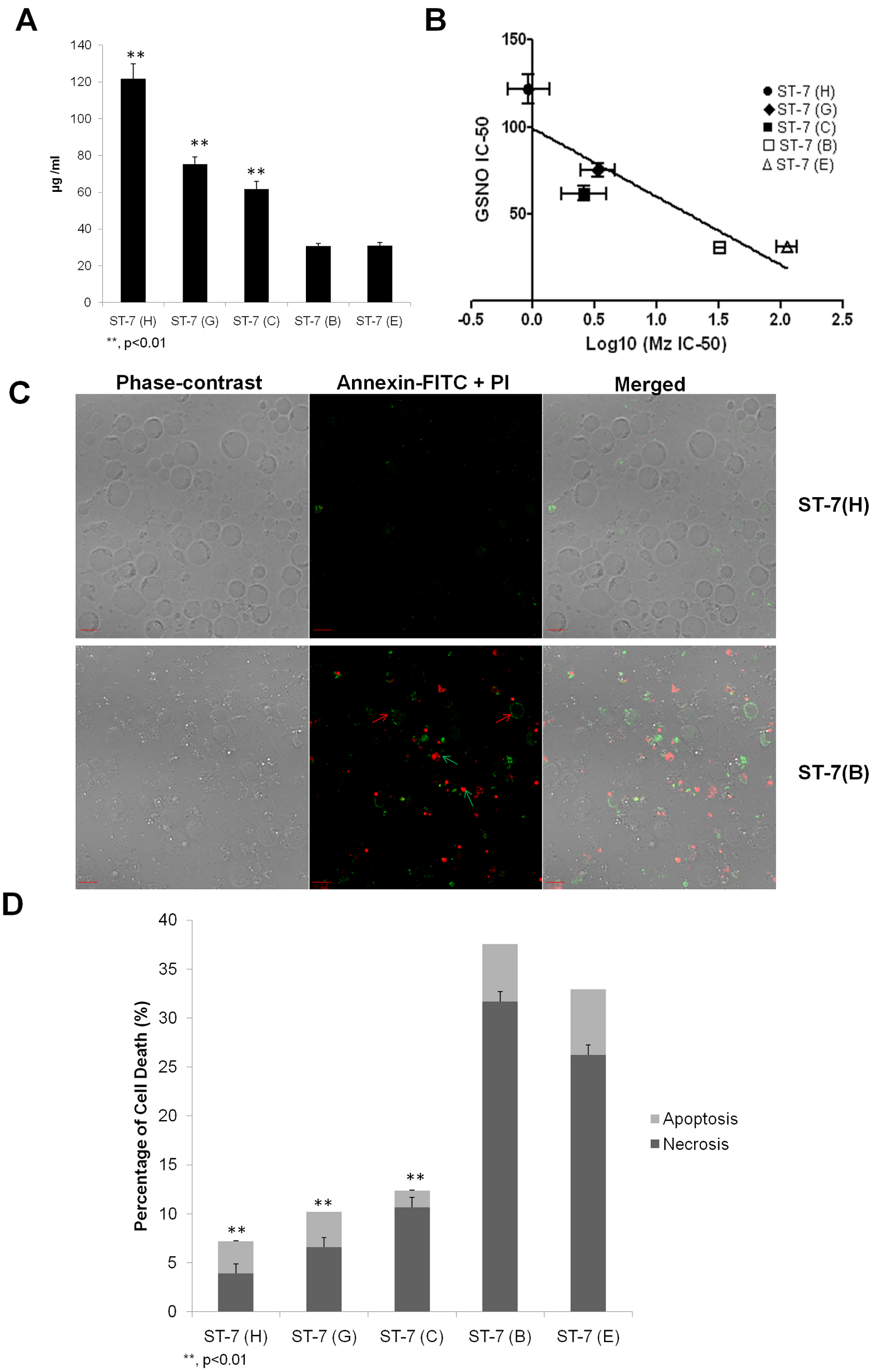

Supplement: Figure S3 — Mz resistant isolates in Blastocystis ST-7 exhibit susceptibility to nitric oxide. (A) Graph representing IC50s of NO donor GSNO against Blastocystis ST-7 strains tested. ST-7 isolates C, G and H showed significantly higher tolerance to GSNO (p<0.01) than Mzr strains B and E. **, p<0.01 vs. ST-7 (B, E). Each point represents a mean of nine readings derived from three independent experiments, triplicate each. The error bar represents standard errors. (B) Relationship between Mz resistance and GSNO resistance in Blastocystis ST-7 parasites. The data points indicate individual strains. Error bars indicate the standard error for the respective measurements (n = 3). There was a negative correlation between level of Mz resistance and tolerance of nitric oxide toxicity (p<0.05, R2 = 0.7972). (C) Confocal micrographs illustrating cell death features of Blastocystis isolates H and B under nitrosative stress. Blastocystis were cultured under culture conditions, in the presence of 50 µg/ml of GSNO. The cells were then stained with Annexin-V-FITC and PI to identify cells undergoing cell death. Features of necrosis (yellow arrows) and programmed cell death (red arrows) were observed. Blastocystis cells undergoing necrosis incorporated both PI and annexin V-FITC stain, cells undergoing programmed cell death, on the other hand bind annexin V-FITC alone. A significantly higher number of dying cells were observed in cultures of ST-7 (B) compared with ST-7 (H) cultures. Scale bar = 10 µm. The micrographs are representative of 15 pictures taken in three separate experiments (five from each). (D) Graph representing percentage of cell death in Blastocystis ST-7 strains after treatment with NO donor GSNO. Compared with Mzs isolates ST-7 (C, G, H), percentages of dead cells in ST-7 (B, E) are significantly higher (p<0.01). **, p<0.01 vs. ST-7 (B, E). Each value represents a mean of nine readings derived from three independent experiments, triplicate each. Error bars represent the standard [file pntd.0002885.s003.tif]

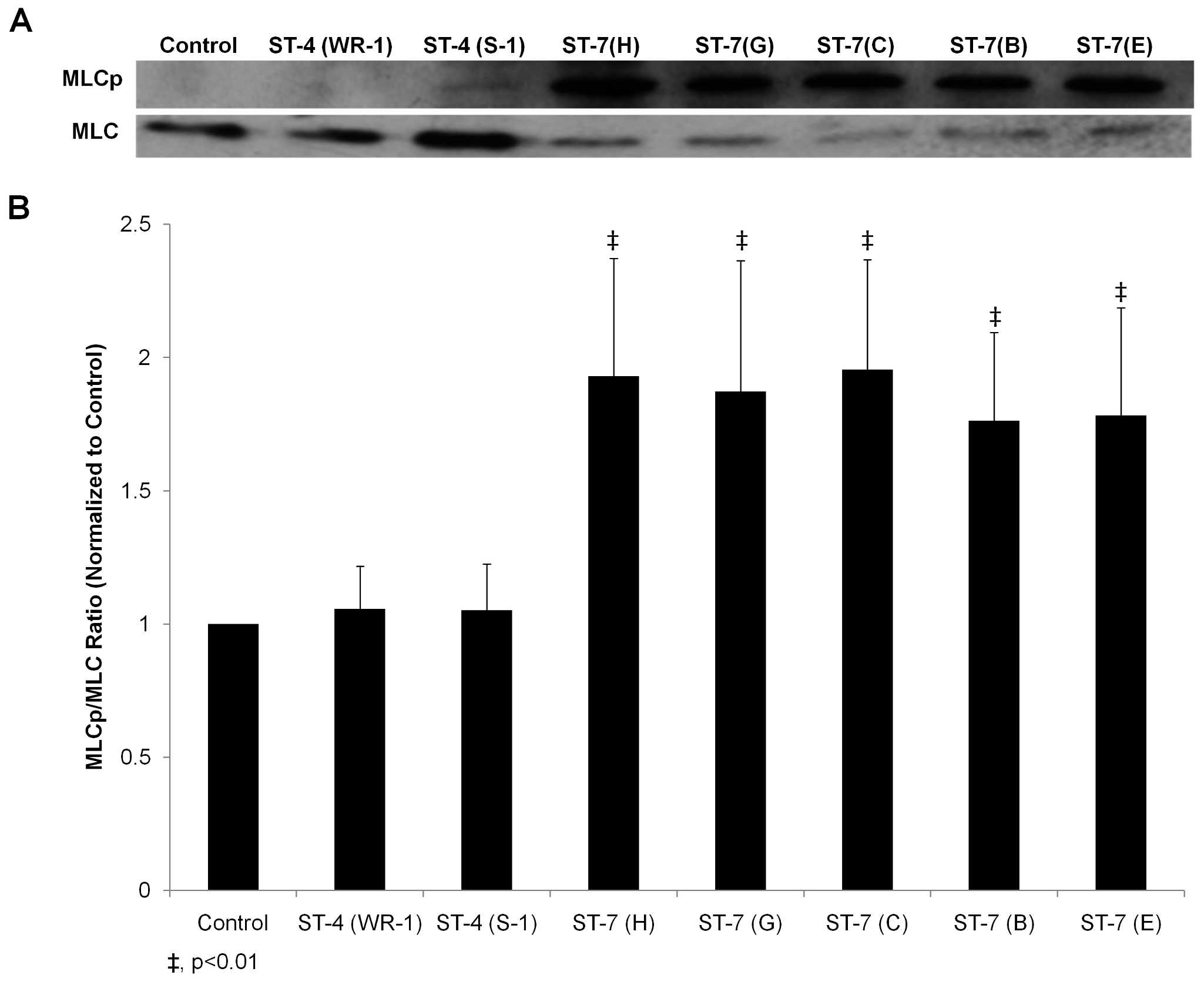

Supplement: Figure S4 — Differential effects on myosin light chain phosphorylation in Caco-2 cell monolayers by different strains of Blastocystis. (A) Representative Western blots exhibiting level of MLCp after incubation with Blastocystis ST-4 and ST-7 parasites. Total MLC was used as a loading control. An obvious increase in MLCp could be noticed in ST-7-treated samples. (B) Graph represents MLCp/MLC ratio normalized to the negative control in Caco-2 cells calculated through densitometry analysis of Western blot radiographs. A significant increase in MLCp was observed in all ST-7-treated Caco-2 epithelium compared with the negative control and ST-4-treated samples (p<0.01). ‡, p<0.01 vs. control. Error bars represent the standard error of the mean of 3 values obtained from three independent Western blots. (TIF) [file pntd.0002885.s004.tif]

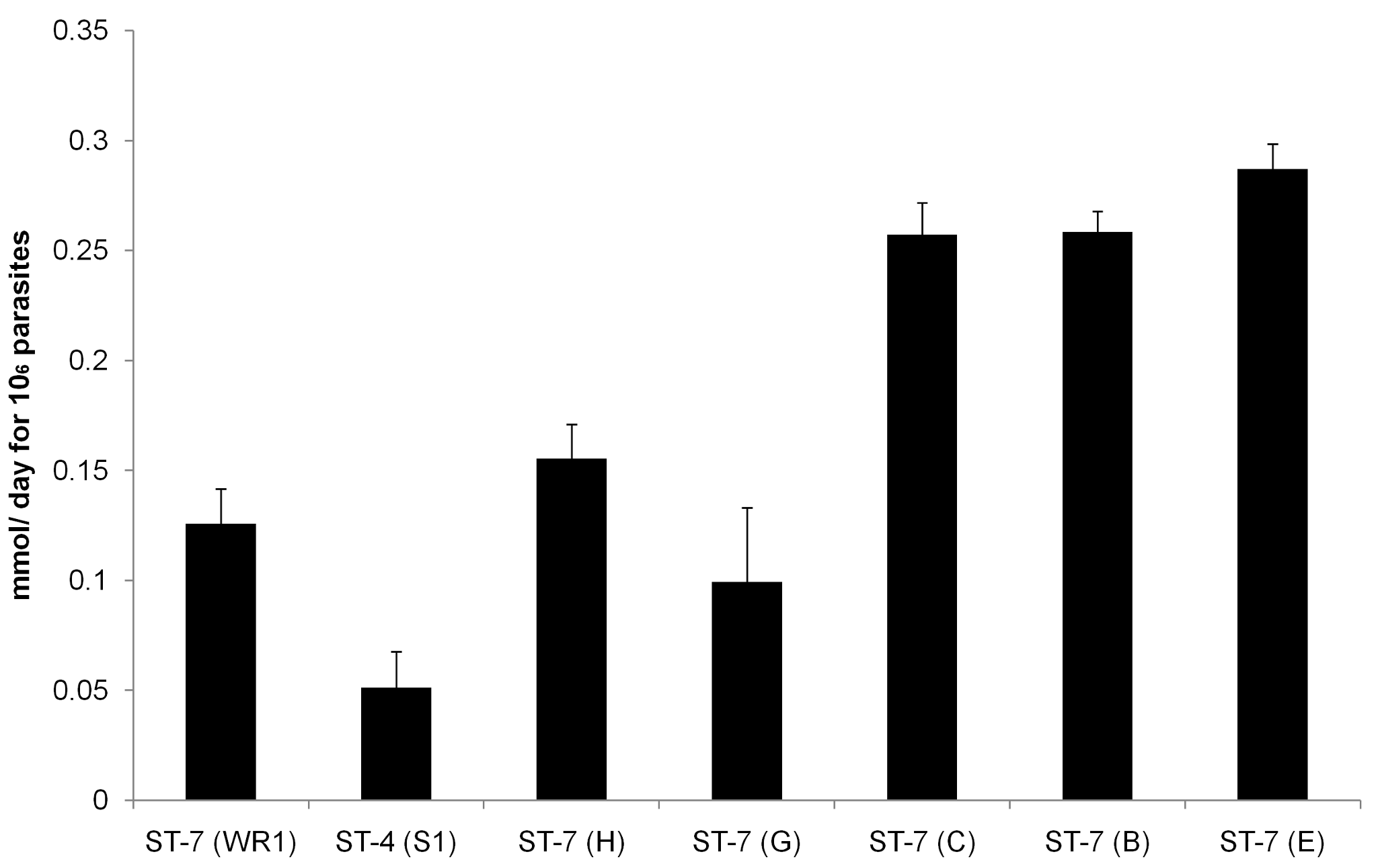

Supplement: Figure S5 — Differential consumption rate of glucose by Blastocystis ST-4 and ST-7 strains. Different strains of Blastocystis from ST-4 and ST-7 were cultured in medium containing 5 mM glucose and incubated under anaerobic conditions, and levels of glucose remaining in the medium were measured after a 24-h incubation period. Glucose consumption was expressed as the mean glucose consumption for 106 cells over the period (mmol/day). No correlation was found between the rate of glucose consumption and the level of attachment or Mz resistance. Each value represents a mean of nine readings derived from three independent experiments, triplicate each. Error bars represent the standard errors. (TIF) [file pntd.0002885.s005.tif]
